# *Lactobacillus reuteri* improves the development and maturation of fecal microbiota in piglets through mother-to-infant microbe and metabolite vertical transmission

**DOI:** 10.1186/s40168-022-01336-6

**Published:** 2022-12-02

**Authors:** Gang Wang, Xinyu Wang, Yonghang Ma, Shuang Cai, Lijie Yang, Yuxin Fan, Xiangfang Zeng, Shiyan Qiao

**Affiliations:** 1 Present Address: State Key Laboratory of Animal Nutrition, College of Animal Science and Technology, China Agricultural University, Beijing, 100193 China; 2 Present Address: Beijing Key Laboratory of Biological Feed Additive, China Agricultural University, Beijing, 100193 China

**Keywords:** Early-life microbiota, Probiotic, Piglets, Multiomic analysis

## Abstract

**Background:**

The immature neonatal fecal microbiota substantially impacts the development of gut health and greatly increases the risk of disease. Developing effective strategies to modulate the development of neonatal fecal microbiota has great significance. Herein, we investigated whether the maternal dietary supplementation and oral administration of *Lactobacillus reuteri* could effectively promote the development and maturation of the fecal microbiome in piglets from birth to weaning.

**Results:**

Metagenomic analysis of colostrum showed that maternal dietary *L. reuteri* supplementation influenced the overall microbiota composition, decreased the abundance of the phylum Proteobacteria and increased that of the species *Bifidobacterium choerinum*. KEGG pathway analysis revealed that maternal *L. reuteri* supplementation enriched the lysine biosynthesis and glycolysis/gluconeogenesis pathways and downregulated the bacterial invasion of epithelial cells in the colostrum. In addition, *L. reuteri* supplementation significantly altered the metabolite features and modules in umbilical cord blood serum based on metabolomics. Further, a significant covariation was observed between these differential metabolites and the species in colostrum. Maternal dietary *L. reuteri* supplementation also significantly influenced the microbiota composition and increased the meconium abundance of beneficial bacteria (such as *Romboutsia*, *Lactobacillus*, *Blautia*, *Butyricicoccus*, and *Ruminococcus*), some of which were markedly associated with several differential metabolites in umbilical cord blood serum between two groups. Notably, both the maternal dietary supplementation and oral intake of *L. reuteri* had strong impacts on the overall microbial composition and maturation of fecal microbiota in piglets during early life, and these effects were dependent on the growth stage. Oral administration of *L. reuteri* promoted diarrhea resistance in neonates, while maternal supplementation of *L. reuteri* enhanced the abilities of antioxidants and decreased inflammation. Moreover, the administration of *L. reuteri* via both methods in combination improved the growth performances of piglets.

**Conclusion:**

Overall, our data demonstrated that *L. reuteri* had the ability to modulate the composition of fecal microbiota in newborn piglets by influencing the microbial community and functional composition in the colostrum and by altering several key metabolites in the umbilical cord blood serum. Also, both the maternal dietary supplementation and oral administration of *L. reuteri* effectively promoted the development and maturation of the fecal microbiome in piglets during early life. Both the maternal dietary supplementation and oral administration of *L. reuteri* in combination optimized the growth performances of piglets.

Video Abstract

**Supplementary Information:**

The online version contains supplementary material available at 10.1186/s40168-022-01336-6.

## Background

The mucosal surface of the mammalian gastrointestinal tract interfaces with a dense and diverse community of microbes, which have been recognized as critical regulators of host physiology and immune homeostasis [1]. In early life, the immune system and intestinal function of newborns are immature, which greatly increases the risk of disease. Infants can receive specific signals from microbes in critical time windows during early development. These microbial signals stimulate the development of the immune system and potentially decrease disease susceptibility [2]. In contrast to its stability and maturity in adults, the microbiota in neonates is less stable and is variable. Increasing evidence has revealed that a dysfunctional early-life microbiota is associated with health outcomes later in life, such as asthma, allergies, obesity, and neuropsychiatric disorders [3–6]. Germ-free mice receiving intestinal microbiota from only neonatal donors contributes significantly to the long-term development of innate and adaptive immunity [7].

The early colonization and maturation of the natural intestinal microbiota are influenced by intrinsic [8] and extrinsic factors [9, 10]. Multiple studies have indicated that oral administration of the commensal infant microbiota via probiotic supplementation is an effective means to improve the early-life dysfunctional microbiota, as observed in human [11] and animal studies [12]. Two double-blinded placebo-controlled randomized clinical trials indicated that postnatal administration of a probiotic mixture to infants could improve microbiota composition and function [13, 14]. The underlying mechanisms of probiotics are hypothesized to be improvement of epithelial barrier function [15], modulation of microbiota composition [16], induction of immunomodulation [17], and suppression of pathogens [18]. In addition to direct regulation of the infant microbiota, maternal factors also influence postnatal early microbiota development. However, infants are sterile at birth, or prenatal colonization by the microbiota remains debated [19, 20]. What is clear is that extensive transfer of the maternal microbiota to the infant could occur during vaginal delivery and breastfeeding, as well as maternal feces [21, 22], resulting in initiation of colonization. The status of the maternal microbiota under the influence of external factors such as diet [23] and infection [24] during pregnancy negatively or positively affects the microbiota in the offspring intestine after birth. Thus, the infant’s intestinal microbiota could be improved by modulating the maternal microbiota that will be transferred to the offspring.

Investigations on the modulation of the development of the infant gut microbiota have mainly focused on the maternal diet [23], feeding style [25], and administered antibiotics [9]. To date, several studies have investigated the relationship of probiotics with the development of gut microbiota in infants [26–29]. However, whether the effect of the probiotic on the mother could modulate the development of infant microbiota through the umbilical cord blood before birth or the milk after birth and whether there is an additive effect of the combination of maternal probiotic supplementation and neonatal oral probiotic administration remain to be determined.

*Lactobacillus reuteri* is a commensal intestinal species that resides in the gastrointestinal tracts of humans and animals [30]. A double-blinded placebo-controlled trial revealed that daily administration of *L. reuteri* to preterm infants modulated the fecal microbiota composition and increased bacterial diversity. Another report also showed that *L. reuteri* promoted the development of the intestinal mucosal system in newborn piglets [31]. *L. reuteri* I5007 (NCBI ID: 1340495) was isolated from the colonic mucosa of healthy weaning piglets in our previous study [32] and was indicated to have several probiotic properties [16, 33, 34]. Pigs are similar to humans in some features, such as microbiology, diet, and genetics and have been used as an animal model in biomedical research [35, 36]. In contrast to rodents, the sow-piglet dyad is a more promising model for the human mother-infant dyad to study the development of intestinal immune systems and the mature of intestinal microbiota during the early postnatal stage of life [37, 38]. Thus, this study aimed to investigate the effect of maternal dietary *L. reuteri* I5007 supplementation on the compositional changes in intestinal and colostrum microbiota in sows and in the fecal microbiota of piglets from birth to weaning. The alteration of metabolite features in the umbilical cord blood serum was also investigated. Our results revealed that *L. reuteri*, administered directly or indirectly via vertical transfer, improved the development and maturation of fecal microbiota and subsequently promoted the early growth of piglets.

## Methods

### Experimental design and sample collection

**Fig. 1:**
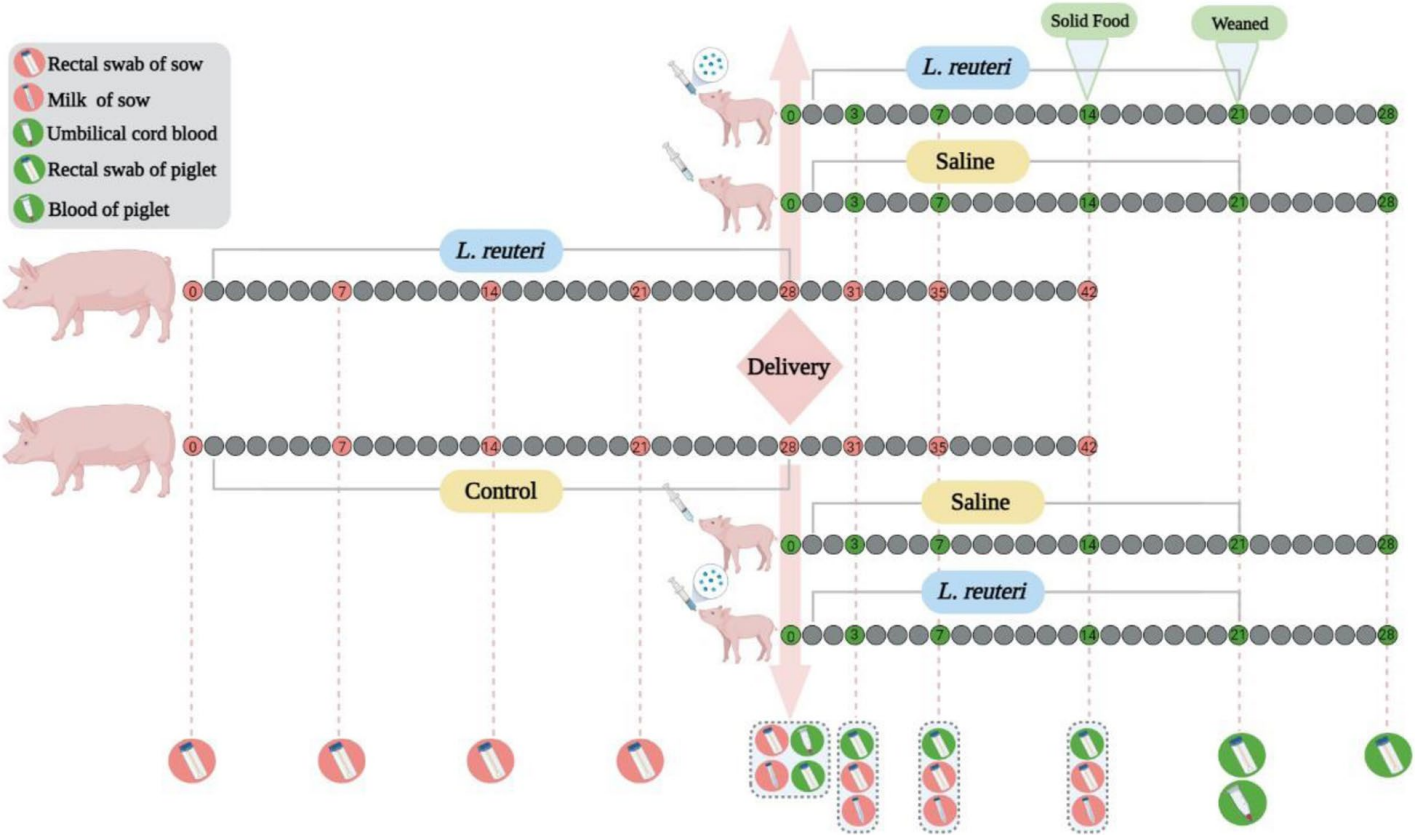
Experimental design and workflow of sample collection. Healthy crossbred sows (Landrace and Yorkshire, *n*=26) in the late gestation stage with similar expected delivery dates were chosen in this study. The sows were randomly allocated into groups with or without the addition of *L. reuteri* I5007 via the diet. The period of treatment in sows was 28 days. After parturition, the piglets from the two groups of sows were divided into two treatments: oral administration of *L. reuteri* I5007 or saline. At day 14 after birth, the piglets were offered creep feed ad libitum and had free access to water. The piglets were kept with the sow until weaning at day 21. The period of treatment in piglets was 21 days. Stool samples of sows and piglets were collected by swabbing. The day when the treatment started was counted as day 0. The figure was created by BioRender.com (https://app.biorender.com/)

Twenty-six healthy sows in late gestation were chosen in this study. The sows were randomly allocated into two groups: control treatment (*n*=13), in which sows were fed the pregnancy diet; I5007 treatment (*n*=13), in which sows were fed the pregnancy diet supplemented with *L. reuteri* I5007 (10^9^ colony-forming units (CFU)/kg diet). The lactation diet fed to the two groups was the same as the pregnancy diet but was not supplemented with *L. reuteri* I5007. After delivery, the number of newborn piglets in each litter per sow was adjusted to six. The piglets were further divided into two treatment groups: oral or nonoral administration of *L. reuteri* in the different sow groups (detail see Fig. 1). Eventually, four groups were generated as follows: (1) Control_Oral group, the piglets from the control group sows with oral administration of *L. reuteri* I5007 (5 mL, 10^9^ CFU/mL); (2) Control_Nonoral group, the piglets from the control group sows with oral administration of sterile saline (5 mL); (3) I5007_Oral group, the piglets from the I5007 group sows with oral administration of *L. reuteri* I5007 (5 mL, 10^9^ CFU/mL); (4) I5007_Nonoral group, the piglets from the I5007 group sows with oral administration of sterile saline (5 mL). After weaning, all piglets remained in nursing pens (six piglets per pen) for another week until day 28. The diet of sows during pregnancy and lactation and the diet of piglets during the suckling period and 1 week after weaning all met the NRC (2012) nutritional requirements.

The rectal swabs of sows were collected on days 0, 7, 14, and 21 during pregnancy, the day of delivery, and days 3, 7, and 14 during lactation. The rectal swab was performed by inserting sterile swab approximately 1–1.5 cm in the anal canal, moving swab from side to side in the anal canal and allowing swab to remain 10–30 s for absorption of microbes onto the swab. After sampling, the swabs were submerged into DNA protection solution and subsequently stored in liquid nitrogen. Umbilical cord blood samples were collected on the day of delivery. The colostrum was collected from each sow within 6 h after delivery. Milk was collected on days 3, 7, and 14 after delivery. To exclude the influence of differences among litters, four litters were randomly selected from each group, and three piglets from each litter were selected for the collection of rectal swabs. Rectal swabs were collected from the piglets from birth to 28 days (days 0, 3, 7, 14, 21, and 28), and blood samples were collected from the piglets on day 21. The serum and colostrum samples were stored at −20 °C, and the rectal swabs were stored in liquid nitrogen after sampling. The body weight of the piglets was recorded on the indicated days to calculate the body weight gain, and the body weight data from piglets from the same litter were averaged to represent the body weight of the litter. The rectal swabs of independent piglets were collected within 1 week of weaning (from days 21 to 28). The fecal samples were from piglets with diarrhea (*n*=16) and from healthy (*n*=16) piglets.

### Preparation of bacteria

*L. reuteri* I5007 was cultured in de Man, Rogosa, and Sharpe (MRS; Solarbio Science and Technology Co. Ltd., Beijing, China) medium at 37 °C for 20 h under anaerobic conditions and then centrifuged at 5000×*g* for 10 min at 4 °C. The pellet was resuspended in reconstituted skim milk (20% w/v) and immediately freeze-dried. The freeze-dried powder containing 10^12^ CFU/g was stored in sealed packets at 4 °C until use. The strain was requantified prior to use. For gavage, *L. reuteri* adjusted to a density of 10^9^ CFU/mL with sterile saline.

### *DNA extraction*, *16S rRNA gene sequencing*, *and data analysis*

Total microbial DNA was extracted from 440 rectal swabs using the QIAamp DNA Stool Mini Kit (Qiagen, Hilden, Germany) according to the manufacturer’s instructions. The extracted DNA was quantified using a NanoDrop (Thermo Fisher Scientific, Wilmington, DE, USA). The V3-V4 hypervariable regions of the 16S rRNA genes were amplified using the universal primers 338F (5′-GTGCCAGCMGCCGCGG-3′) and 806R (5′-CCGTCAATTCMTTTRAGTTT-3′). The optimized conditions for PCR amplification were as follows: initial denaturation for 3 min at 95 °C; 27 cycles of 30 s at 95 °C, 30 s at 55 °C, and 45 s at 72 °C; and a final elongation step for 10 min at 72 °C [39]. Two-percent agarose gel electrophoresis was performed to confirm the size of the PCR products. Then, the amplicons were quantified, pooled, and sequenced using the Illumina MiSeq system (Illumina Inc., San Diego, CA, USA) for paired-end reads. Sequence analysis was performed using the UNOISE pipeline through USEARCH v10.0 [40] and VSEARCH v2.15 [41]. Briefly, paired-end sequences were merged, quality filtered, and dereplicated using VSEARCH; denoising and chimera removal were then conducted using UNOISE3 to correct for sequencing errors. Assembled sequences were mapped back to the denoised chimera-free sequences as OTUs with 97% identity. The taxonomy of the features was classified by the USEARCH SINTAX algorithm in RDP training version 16 (http://rdp.cme.msu.edu/) [42]. The average V3-V4 16S rRNA gene sequencing depth was 43,559 ±7824 reads. The sequences of all the samples were downsized to 27,226 to match the difference in sequencing depth. α-diversity was measured from the rarefied OTU table by the richness, Simpson, and Shannon indices. β-diversity was estimated by Bray–Curtis distance, which were applied to build distance matrices and reported according to principal coordinate analysis (PCoA). For differential abundance analysis and association analysis, the taxa with relative abundance <0.01% and present in <20% across all samples were removed.

### DNA extraction, metagenomic sequencing, and data analysis

Total microbial genomic DNA was extracted from 16 colostrum samples (1 mL) using the Milk DNA Extraction Kit (Norgen, Thorold, Ontario, Canada) following the manufacturer’s instructions. The quantity and quality of the extracted DNA were measured using a NanoDrop (Thermo Fisher Scientific, Wilmington, DE, USA) and agarose gel electrophoresis, respectively. The extracted microbial DNA was processed to construct metagenome shotgun sequencing libraries with insert sizes of 400 bp by using the Illumina TruSeq Nano DNA LT Library Preparation Kit.

The raw sequencing reads were first quality-controlled by Trimmomatic [43], and the clean reads were then aligned to the pig (*Sus scrofa*) genome with Bowtie2 [44] to exclude host contamination. Quality-filtered metagenomic reads were taxonomically profiled using Kraken2 [45]. High-quality reads were de novo assembled into contigs using MEGAHIT [46], generating a total of 364,772 contigs with an N50 length of 878 bp. The genes were annotated using Prokka (v.1.13.3) [47]. Then, the metagenomic contigs of all the samples were clustered by CD-HIT (v 4.5.8) [48] to obtain unigenes, and the abundance of unigenes in each sample was estimated by calculating the TPM value based on the number of aligned reads using Salmon (v 0.9.1) [49]. To identify metagenomic functional categories, the protein sequences were annotated against those of evolutionarily conserved genes in the nonsupervised orthologous groups (eggNOG) and CAZy databases using DIAMOND (v.0.8.23) [50] with default parameters. Statistically significant differences in taxa and functions across groups were determined by linear discriminant analysis (LDA) effect size (LEfSe) with the default parameters [51]. An LDA score >2.0 and *p*<0.05 were used as the thresholds for significant differences in the taxa and functions.

### Untargeted metabolomic study

Metabolite concentrations in umbilical cord blood serum were quantified using UPLC-TOF-MS in both positive and negative ionization modes. Twenty microliters of a sample was extracted with 120 μL of cold methanol/acetonitrile (1:1, v/v) and vortexed for 1 min. The mixture was homogenized for 5 min and subsequently centrifuged at 14,000×*g* for 15 min at 4 °C. In addition, pooled QC samples were also prepared by combining 10 μL of each extraction mixture. Metabolite profiling was performed by using the procedures described previously [52].

The acquired data pretreatments included the retention time, m/z, and peak area of each sample. Each ion was identified based on the retention time and the m/z data pairs. Then, we obtained the intensities of each peak and a matrix including arbitrarily assigned peak indices (retention time-m/z pairs), sample names (observations), and ion intensity information (variables). The matrix was further reduced by removing peak data with missing values in more than 80% of the biological samples or 50% of the QC samples. After normalization to the total peak intensity, the processed data were uploaded into MetaboAnalyst software for further analysis (www.metaboanalyst.ca). Orthogonal projection to latent structure discriminant analysis (OPLS-DA) was performed to discriminate among the different metabolites between groups. Only metabolites with variable importance in the projection (VIP) greater than 1.0 were further analyzed using Student’s *t* test at the univariate level with adjusted *P* < 0.05.

### Clustering of coabundant metabolites

Clustering of coabundant metabolites was performed using the R package weighted correlation network analysis (WGCNA) [53] based on correlation. The correlation matrix quantified interconnectedness between metabolites and assigned them to coabundance modules. The metabolites that did not show high enough coexpression metrics with any module were removed. The signed network was derived based on a biweight midcorrelation (bicor) method. A scale-free topology criterion was used to choose the soft threshold of eight for metabolite correlations. The minimum module size was five.

### Determination of SCFA concentrations

The concentrations of short-chain fatty acids (SCFAs), including formic, acetic, propionic, butyric and lactic acid, in sow milk, and umbilical cord blood serum, were determined as previously described with slight modifications [54]. Briefly, 100 μL of milk or serum was added to 400 μL of methanol/acetonitrile (1:1, v/v) for protein precipitation, and the mixture was vortexed for 2 min and kept at room temperature for 10 min. The samples were then centrifuged at 12,000 rpm and 4 °C for 20 min, and the supernatant was transferred into an Eppendorf tube. The supernatant was then diluted with ultrapure water, vortexed for 1 min, and filtered through a 0.22-μm filter. The SCFA samples were placed in a 2-ml screw-cap vial and identified by high-performance ion chromatography (ICS-3000; Dionex, Sunnyvale, CA, USA) with a conductivity detector. Finally, the concentrations of the SCFAs were calculated from the peak areas of the compounds relative to the internal standards.

### Interspecies correlations in each group

The species that showed differences in abundance (LEfSe: *P*<0.05, LDA>2) between the two groups across all colostrum samples were considered key species. Then, the sparse correlations among the key species were determined in the SparCC [55] python module with a bootstrap repeated 100 times. Co-occurrence network properties were analyzed with the R package igraph [56]. Only the correlation scores with absolute values greater than 0.7 and *P* values less than 0.05 were visualized by Cytoscape (v 3.7.1). To describe the topology of the network, several parameters (e.g., node number, edge number, clustering coefficient, average path length, average degree, graph density, betweenness centrality, degree centrality) were calculated [57]. We assessed the difference between species networks by bootstrapping node attributes (node degree, closeness centrality, betweenness centrality, and transitivity centrality) using a two-sample Kolmogorov–Smirnov test (bootstrap *n*=10,000). The Kolmogorov–Smirnov test compares the overall shape of the cumulative distribution of two variables, where the null hypothesis is that the variables have the same distribution patterns [58].

### Determination of microbiota maturity using random forest

Age-specific taxa were identified with distinctive time-dependent changes in their relative abundances, and then, a random forest machine learning algorithm was used to regress the OTUs in the time-series profiles of the fecal microbiota against their chronologic age using default parameters of the R package randomForest [59]. Since the random forest algorithm has nonparametric assumptions, it was applied to detect both linear and nonlinear relationships between OTUs and chronological age, thereby identifying taxa that were highly correlated with periods of postnatal gut microbiota development. The random forest model was then applied to test the OTUs in the *L. reuteri* group. For the training of the random forest model, a smoothing spline function was fitted between microbiota age and chronological age of the control group. Based on the model, relative microbiota maturity was used to define the maturation of the fecal microbiota. The metrics were calculated as described previously [60]. The differences in microbiota maturation metrics among chronologic ages in the two groups were analyzed using the Wilcoxon rank-sum test.

### Coinertia analysis (CIA) between the microbiota and metabolite data sets

Coinertia analysis is a method for ordination of the costructure between two data sets depending on their linear combinations. Additionally, CIA is coupled with principal component analysis or correspondence analysis. In the case of CIA, the coinertia between two data sets is maximized and decomposed. In this study, we used CIA to identify the relationship between the microbiota and serum metabolite data sets using the R package ade4 [61]. Two independent PCoAs were conducted based on the microbiota composition and serum metabolites using the relative abundance and Bray–Curtis distance and then subjected to coinertia analysis. The overall similarity between two data sets was then measured by the regression vector (RV) coefficient [61]. A Monte Carlo test was used to measure the significant association between two data sets.

### Weight gain, diarrhea incidence, inflammatory cytokines, and antioxidant index

Piglets were weighted on days 0, 7, 14, 21, and 28. The average daily gain (ADG) was calculated on a litter basis. To evaluate the incidence of diarrhea, the fecal consistency of piglets was visually assessed three times per day during the experimental period by fixed observers blinded to the treatment according to the method described previously [62]. The diarrhea incidence was calculated as follows: diarrhea incidence (%) = (number of piglets with diarrhea) / (number of piglets × total observational days) × 100. The levels of interleukin 6 (IL-6), tumor necrosis factor-α (TNF-α), and malondialdehyde (MDA) and the activity of superoxide dismutase (SOD) in the serum were measured using the corresponding commercial kits (CUSABIO, https://www.cusabio.com/) according to the manufacturer’s protocol.

### Diarrhea classification based on fecal microbiota profiles by a support vector machine (SVM)

The health condition of each piglet was evaluated daily over the 28-day experimental period. Since diarrhea may cause temporal changes in the diversity and stability of the fecal microbiota and last several days, we only collected samples at the indicated times. If diarrhea occurred at a nonsampling time point and was continuous for 2 days, the sample was assigned to the next sampling point. For example, if a piglet exhibited diarrhea on days 15 and 16, the sample from the piglet was classified as “diarrhea” on day 21. To determine whether properties of the fecal microbiota could be used as biomarkers to distinguish between healthy and diarrheic animals, we built a machine learning model by SVM as previously described [63]. To establish the SVM model for predicting diarrhea, the minimal redundancy maximal relevance (mRMR) method and leave-one-out cross-validation (LOOCV) were applied. The OTU set with the highest Matthews correlation coefficient (MCC) was chosen as the final biomarker and to build the SVM classifier. The test set and another independent cohorts were used to assess the predictive power of the classifier based on the receiver operating characteristic (ROC) curves in the R package pROC [64].

### Statistical analysis

GraphPad Prism (v 6.0) and the R program (v 3.6.1) were used for statistical analysis. PCoA with Bray–Curtis distance was used to visualize the phylogenetic similarities across samples. Permutational multivariate analysis of variance (PERMANOVA) (“adonis” function, R package vegan [65]) was applied to explore differences in the microbial or functional composition in terms of β-diversity with 999 permutations. The differences in α-diversity (richness, Shannon, and Simpson) indices, within-group Bray–Curtis distance and the relative abundance of taxa were calculated using the Wilcoxon rank-sum test from sow 16S rRNA data. Redundancy analysis (RDA) was performed at the genus level using the R package vegan. Differences in the relative abundance of the taxa and functional features (KO, GO, and CAZy orthologs and KEGG pathways) from metagenomic data of colostrum samples were determined by LEfSe [51] using the online Huttenhower Galaxy server (http://huttenhower.sph.harvard.edu/galaxy/). A replicate (litter) was considered the experimental unit for analysis of differences in weight gain and diarrhea incidence, which were analyzed by two-way ANOVA. During statistical testing of the α-diversity parameters, the Bray–Curtis distances within groups and differences in the microbial features of piglets were examined by the Scheirer-Ray-Hare test (a nonparametric alternative to two-way ANOVA) with the R package rcompanion [66]. Correlations between metabolites or serum metabolite modules and the relative abundance of species from metagenomic data were tested with the Spearman rank. Spearman’s rank correlation coefficients were calculated using the cor.test function and visualized with a heatmap constructed by the R package pheatmap [67]. The differential abundance of modules was tested by the Wilcoxon rank-sum test. Other figures, such as scatter plots, box plots, histograms, and line charts, were generated by the ggplot2 package [68]. All *p* values of multiple comparisons in the above analysis were adjusted to the false discovery rate (FDR) using the Benjamini-Hochberg (BH) method except where noted. The threshold of significant difference was set at *P*<0.05 (*P<0.05, **P < 0.01, ***P < 0.001, ****P < 0.0001), and *P* < 0.1 was considered a significance trend.

## Results

### Compositional changes in the fecal microbiota of sows

Twenty-six crossbred sows (Landrace and Yorkshire) in late gestation received a diet supplemented with *L. reuteri* I5007 from late gestation (days 0–28) to lactation (days 28–42). We first investigated the changes in fecal microbiota composition after I5007 treatment. No significant differences in microbial richness and diversity (Shannon and Simpson index) were found in the intestine across different ages between the two groups (Additional file 1: Fig. S1A). PCoA based on Bray–Curtis distance was used to assess the overall structure of the fecal microbiota, and the results showed that the addition of I5007 did not change the composition of the microbiota visibly (Additional file 1: Fig. S1B). Similar results were observed for the interindividual Bray–Curtis metrics (Additional file 1: Fig. S1C). The α- and β-diversity values of the fecal microbiota showed clear age-dependent progression over the entire experimental period.

At the phylum level, Bacteroidetes, Proteobacteria, and Firmicutes were the most abundant taxa in the fecal microbiota of sows over time, and the relative abundance of Proteobacteria tended to increase from gestation to lactation (Additional file 1: Fig. S1D). There were several significantly different genera at different ages between the two groups (Wilcoxon test, *P* value <0.05). Among these genera, *Anaerostipes* at day 14 (Additional file 1: Fig. S1E), *Lachnospiracea_incertae_sedis* and *Mogibacterium* at day 28, *Ruminococcus* at day 35, and *Acinetobacter* at day 42 exhibited significantly higher relative abundances in the I5007 group than in the control group. These results indicated that *L. reuteri* I5007 may influence only specific bacterial taxa at a specific age but not the overall community structure in the fecal microbiota of sows.

### Alterations in the metabolomic profile of the colostrum

**Fig. 2:**
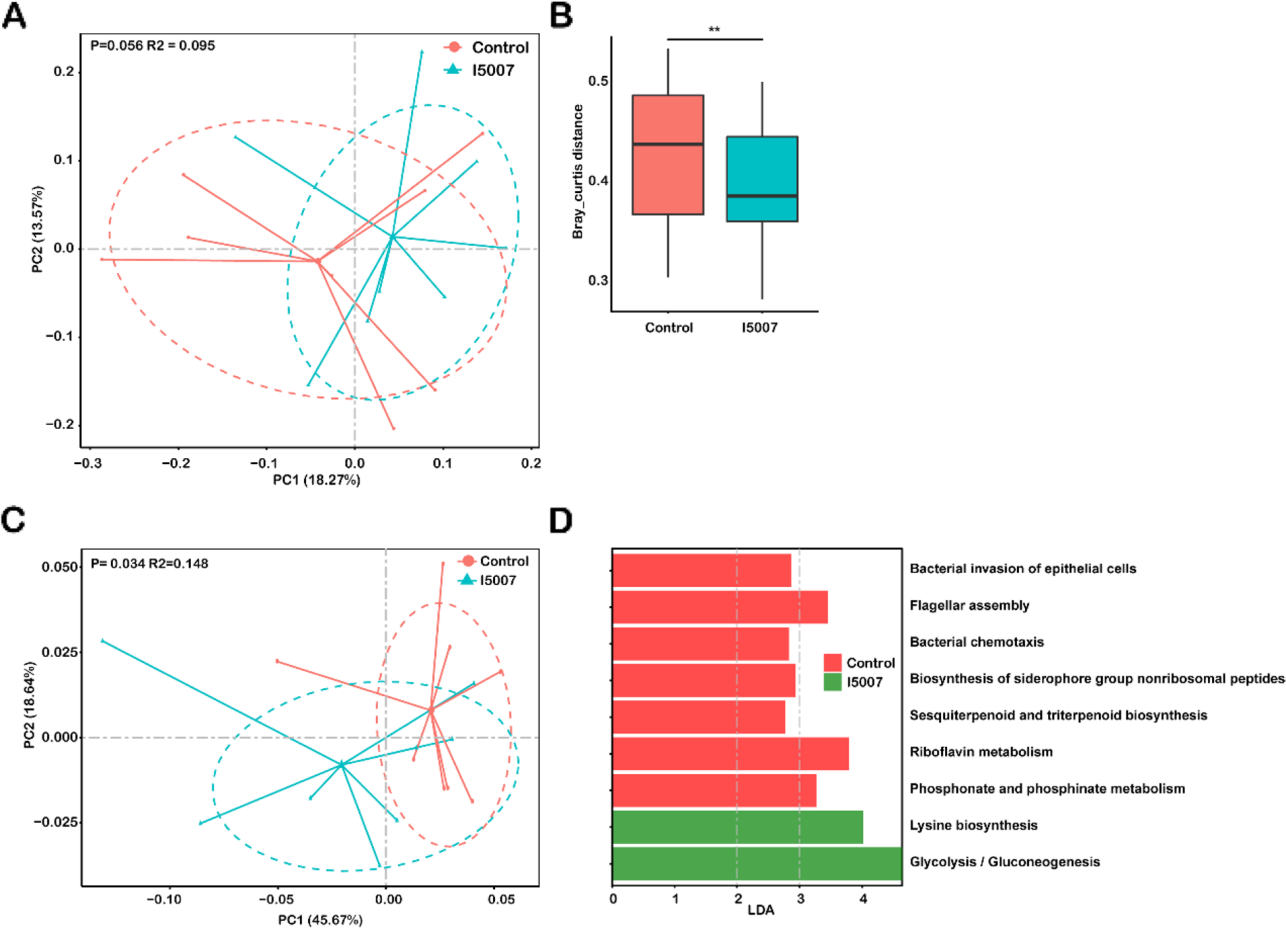
Alterations in microbial and functional compositions in the colostrum**. A** A principal coordinate analysis (PCoA) with the Bray–Curtis distance was performed to assess the community structure of all the species’ relative abundances from the metagenome. **B** The microbial structure dissimilarity was dependent on the Bray–Curtis metric between two groups. The Wilcoxon rank-sum test was used to analyze variation between two groups. **P<0.01. **C** PCoA plots of all GO terms based on the Bray–Curtis distance. **D** Histograms of significantly enriched KEGG pathways between the I5007 and control groups based on the criteria of an LDA>2 and *P*<0.05

In addition to providing nutrition, breast milk contains a diverse microbiota and active components that are thought to be important for the development of the infant immune system and microbiome [69]. Whether the maternal dietary supplementation of *L. reuteri* affects the microbial communities and functional composition of colostrum remains unknown. To determine these effects, we performed whole-genome shotgun sequencing of colostrum samples. In contrast to the fecal microbiota composition of sows, the taxonomic profile showed that the 3 most abundant phyla in the colostrum were Actinobacteria, Firmicutes, and Proteobacteria, and the 5 most abundant families were Corynebacteriaceae, Lactobacillaceae, Streptomycetaceae, Burkholderiaceae, and Staphylococcaceae (Additional file 1: Fig. S2A and S2B). Maternal dietary *L. reuteri* intake increased the abundance of Actinobacteria (37.4% → 47.40%) and decreased that of Proteobacteria (28.03% → 19.19%) (Additional file 2: Table S1a). We further examined the influence of a dietary *L. reuteri* on the overall microbiome composition in colostrum samples by performing PERMANOVA with the Bray–Curtis distance (Fig. 2A). The *L. reuteri* supplement tended to alter the species composition (*P*=0.056) and explained 9.5% of the interindividual variation. The within-group Bray–Curtis metric was significantly lower in the I5007 group than in the control group (*P*=0.0045) (Fig. 2B). The α-diversity analysis of the milk microbiota showed that there was no obvious alteration among these indices between the two groups (Additional file 1: Fig. S2C).

**Fig. 3:**
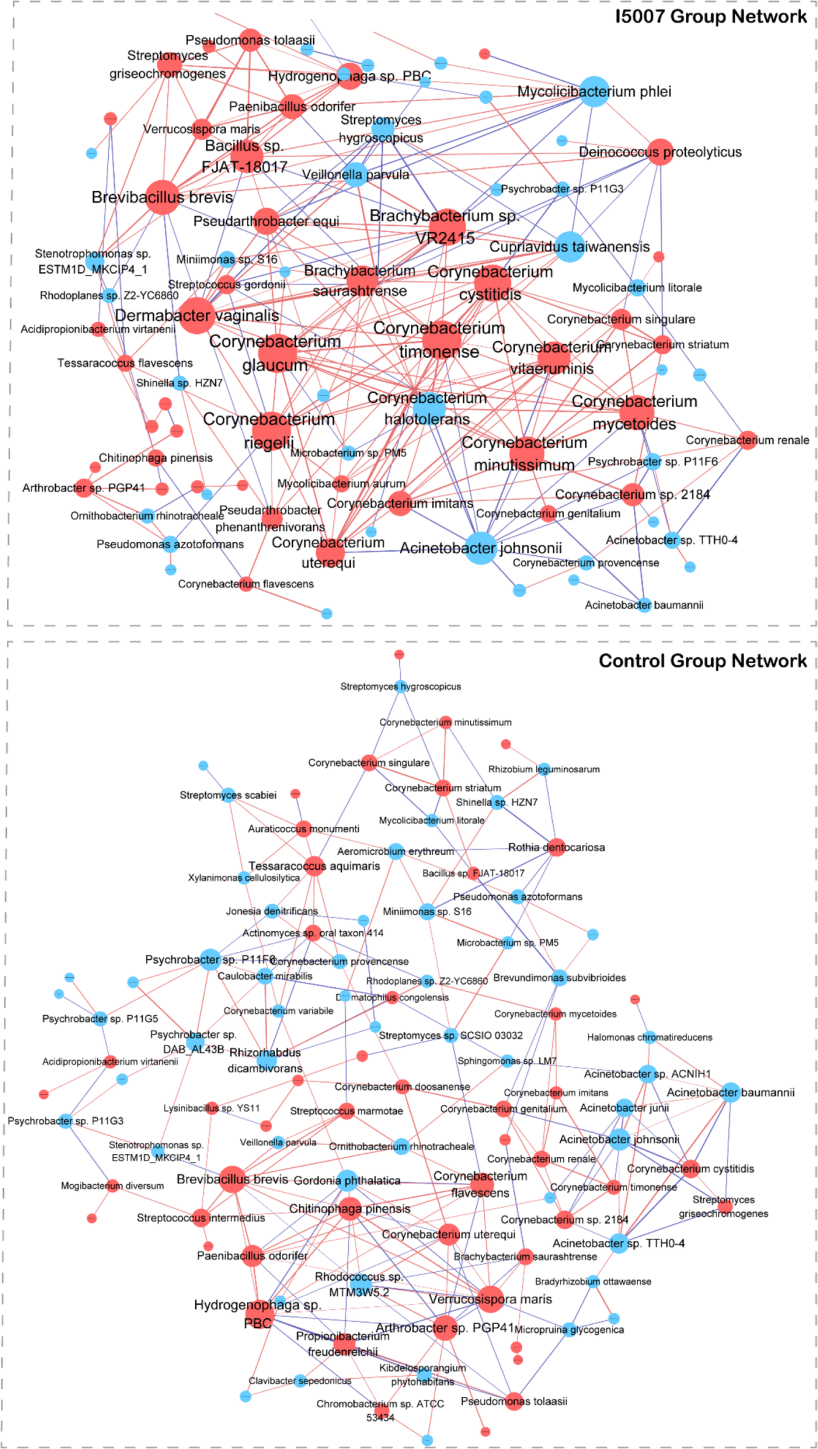
Co-occurrence network of the significantly different species. Edges representing significant SparCC correlations indicate |*r*|>0.7 and *p*<0.05. Light blue lines represent a significant negative correlation, and light red lines represent a significant positive correlation. The size of the points represents the degree of the node. The thickness of the line is proportional to the value of correlation. The node color represents the enrichment of the species in the I5007 (red) and control (blue) groups

A total of 112 species showed changes in abundance upon the administration of I5007 with the criteria LDA>2.0 and *P*<0.05 (Additional file 1: Fig. S3; Additional file 2: Table S1b). Fifty-six of the 112 species were enriched in the I5007 group, which were mostly from the phyla Actinobacteria and Firmicutes, whereas the major species enriched in the control group were of the phylum Proteobacteria. Notably, we also observed that dietary *L. reuteri* supplementation significantly increased the abundance of *Bifidobacterium choerinum*. PCoA of the relative abundances of differential species based on the Bray–Curtis distances enabled clear separation of the I5007 and control groups and explained 20.2% of the interindividual variation (Additional file 1: Fig. S2D). To describe the interbacterial patterns of interactions within the milk microbiome, we constructed co-occurrence networks of these altered species from each group based on significant correlations (*P*<0.05, |R|>0.7) (Fig. 3). The results revealed a more complex network of the I5007 microbial features. The I5007 and control groups shared 23 overlapping edges, while 221 and 189 edges were specific to the I5007 and control groups, respectively. Further insight into the feature network suggested that the I5007 microbial feature network had a higher clustering coefficient, average degree, graph density, betweenness centrality, and degree centrality than that of the control group (Additional file 2: Table S1c). The analysis of the difference between the two networks by bootstrapping node attributes (node degree, closeness centrality, betweenness centrality, and transitivity centrality) using the Kolmogorov–Smirnov test also showed that all these features were significantly different between the two groups (*P*<0.0001). Notably, there were 19 species and 4 species with more than 10 nodes in the networks of the I5007 and control groups, respectively (Additional file 2: Table S1d and S1e).

To characterize the functional changes in the microbial community in milk after I5007 treatment, we annotated the metagenomic gene catalogs against the KEGG, GO, and CAZy databases. The PERMANOVA test based on Bray–Curtis distance revealed that the dietary *L. reuteri* had a significant effect on the functional structure based on GO terms (Fig. 2C). LEfSe (LDA>2.0, *P*<0.05) revealed that 70 KOs were significantly changed between the two groups (Additional file 2: Table S1f). Forty-four of the 70 KOs were aligned to KEGG pathways and were mainly involved “metabolism,” “genetic information processing,” and “environmental information processing.” Nineteen of the KOs were aligned to protein families, which were mainly involved in “genetic information processing” and “signaling and cellular processes.” Pathway enrichment analysis revealed that 9 KEGG pathways were significantly different between the two groups (Fig. 2D). The upregulated KEGG pathways in the I5007 group included lysine biosynthesis and glycolysis/gluconeogenesis, whereas the major KEGG pathways enriched in the control group were linked to several bacterial environmental responses (e.g., bacterial invasion of epithelial cells, bacterial chemotaxis, biosynthesis of siderophore group nonribosomal peptides, and flagellar assembly), and these were only positively correlated with the differentially abundant species in the control group (Additional file 1: Fig. S4). PCoA showed that there was no statistically significant difference among CAZyme profiles using the Bray–Curtis distance between the two groups (Fig. S5A). We also measured the concentrations of several metabolites produced by microbial metabolism. The results showed that maternal dietary *L. reuteri* intake significantly influenced the level of lactate in the colostrum (Additional file 1: Fig. S5B). The concentrations of both lactate and propionate changed depending on age (Additional file 1: Fig. S5B and S5C). There were no differences in other SCFAs (data not shown).

### Changes in the metabolite features of umbilical cord blood serum

**Fig. 4:**
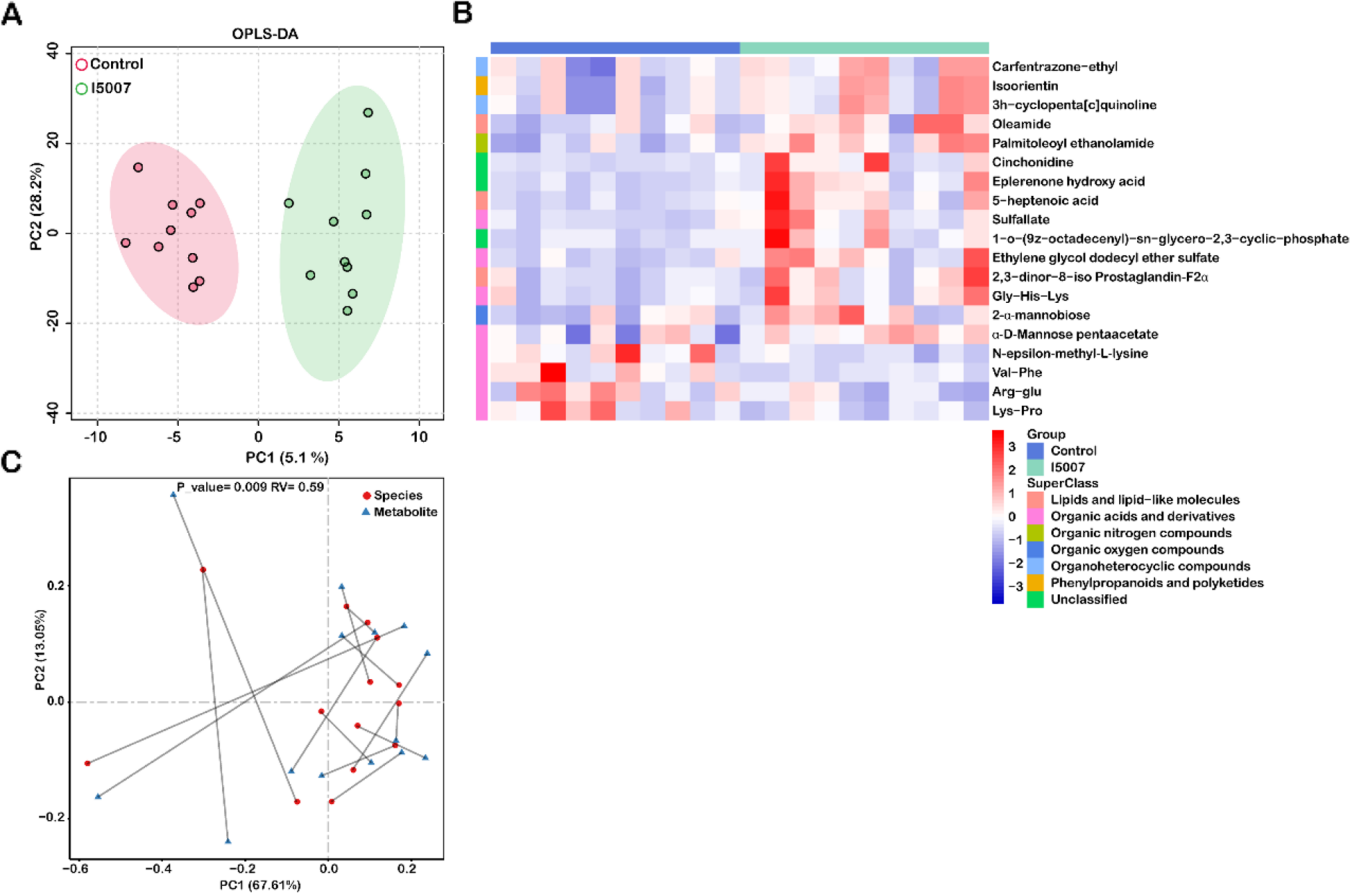
Alterations in the serum metabolite features and the relationship with the microbiota of sows. **A** Scatter plot of orthogonal projection to latent structures discriminant analysis (OPLS-DA) results for the serum metabolome across the two groups. **B** Heatmap showing the levels of 13 significantly different serum metabolites between the I5007 and control groups. Metabolites meeting the criteria VIP>1 and *P*<0.05. **C** Coinertia analysis of the relationship between 13 differential serum metabolites and 112 differentially abundant species in the colostrum. PCs represent the two first principal components from the CIA. The differences were analyzed by the Monte Carlo test

The metabolic products derived from the microbiota and host could enter the bloodstream and play an important role in host physiology. As we observed that dietary administration of *L. reuteri* modulated the colostrum microbiome, we wanted to determine whether the metabolite features of umbilical cord blood serum were also affected by *L. reuteri.* We further explored the untargeted serum metabolome profiles via UPLC-TOF-MS in both the positive ion mode (ES+) and negative ion mode (ES−). We identified a total of 1087 metabolites after eliminating impurity peaks and removing repetitive ions in both modes for further analyses. Based on the OPLS-DA models of all the metabolite profiles, we found that there was a clear separation between the two groups (Fig. 4A). Based on the criteria of a VIP>1 and *P*<0.05, we identified 19 metabolites that were significantly altered by supplementation with *L. reuteri*. Fifteen of the 19 metabolites were enriched in the I5007 group, and these metabolites could be grouped into organoheterocyclic compounds, phenylpropanoids and polyketides, lipids and lipid-like molecules, organic acids and derivatives, organic nitrogen compounds, and organic oxygen compounds. The other 4 metabolites were enriched in the control group, and all of these metabolites were grouped into organic acids and derivatives (Fig. 4B). In addition to nontargeted metabolomic profiling, we also examined the concentrations of SCFAs, including formate, acetate, propionate, lactate, butyrate, isobutyrate, and valerate in umbilical cord blood serum, but no difference was found between the I5007 and control groups (data not shown).

In complementary analyses, we classified these 1087 serum metabolites into 25 different coabundance modules (excluding the gray module) of closely associated metabolites based on WGCNA (Additional file 2: Table S1g). By abundance comparison, we found that three modules differed between the I5007 and control groups (Additional file 1: Fig. S6). Several metabolites in the MEblack (2-α-mannobiose), MElightcyan (2,3-dinor-8-iso prostaglandin-Fα, Gly-His-Lys, Arg-Glu), and MEpink (sulfallate, ethylene glycol dodecyl ether sulfate, eplerenone hydroxy acid, 5-heptenoic acid and 1-o-(9z-octadecenyl)-sn-glycero-2,3-cyclic-phosphate) modules also overlapped with those identified as differential metabolites between the two groups above (Fig. 4B).

Next, we investigated whether covariation existed between the serum metabolites and microbiota in sows, and the CIA approach followed by a Monte Carlo test was used to assess the association between two data sets. The differential species in the colostrum were significantly associated with the altered metabolites (Fig. 4C). Moreover, the RV coefficient was lower and the *p* value was higher when comparing the CIA results of the altered species and all serum metabolites, which further indicated covariation between the markedly different metabolites and the differential microbiota (Additional file 1: Fig. S7A). In addition, no association was observed between the serum metabolites and fecal microbiota (Additional file 1: Fig. S7B and S7C).

### Dietary supplementation and oral administration of L. reuteri both modulated the fecal microbiota in piglets

**Fig. 5:**
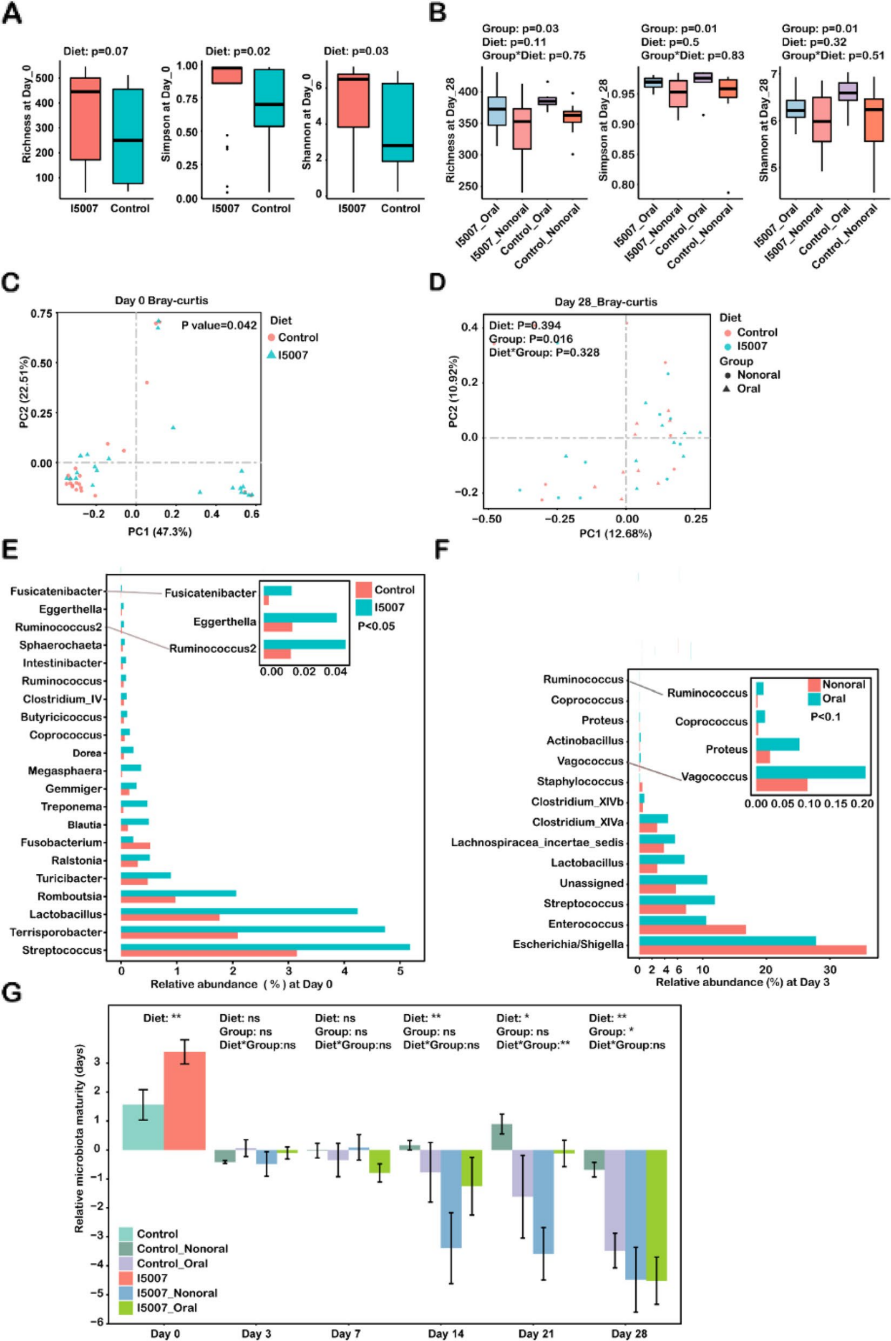
Modulatory effect of *L. reuteri* on the fecal microbiota in piglets. **A** Boxplots of *α*-diversity (richness, Shannon, and Simpson index) at day 0. The median of the data is shown. The Wilcoxon rank-sum test was used to analyze the variation between two groups. **B** Boxplots of α-diversity (richness, Shannon, and Simpson index) at day 28. Differences were analyzed by two-way ANOVA based on the Scheirer-Ray-Hare test. PCoA with Bray–Curtis distance was performed to assess the microbiota structure at **C** day 0 and **D** day 28. **E** Bar graph showing significant differentially abundant genera between the *L. reuteri* and control groups at day 0. The data are expressed as the average relative abundance of genera in each group. The Wilcoxon rank-sum test was used to analyze the variation between two groups. **F** Bar graph of significant differentially abundant genera between the Oral and Nonoral groups at day 3. **G** The effect of maternal dietary supplementation and oral intake of *L. reuteri* on relative microbiota maturity at different time points. The Wilcoxon rank-sum test or two-way ANOVA was used to analyze the variation. ns, not significant; *P <0.05; **P<0.01; ****P <0.0001. The data are expressed as the mean ± SEM. Diet indicates dietary supplementation with *L. reuteri* or not; group indicates the oral administration of *L. reuteri* or not

We further examined whether maternal dietary *L. reuteri* supplementation influenced the fecal microbiota composition of piglets from birth (day 0) to day 28. Analysis of microbiota composition by 16S sequencing indicated that dietary *L. reuteri* supplementation increased the Shannon (*P*=0.02) and Simpson (*P*=0.03) indices in the meconium of newborn piglets, and an increasing trend was observed for richness (*P*=0.07) (Fig. 5A). A similar result was observed for the PCoA based on the Bray–Curtis distance at the OTU level, and dietary *L. reuteri* regulated the overall microbial structure (*P*=0.042) (Fig. 5C). To explore in detail the change in the microbiota, we analyzed the relative abundances of the genera that were different between the two groups. Compared to the control group, dietary *L. reuteri* supplementation also highly enriched the abundances of 21 bacterial taxa at the genus level at day 0, such as *Romboutsia* (0.97% → 2.06%), *Lactobacillus* (1.77% → 4.24%), *Blautia* (0.12% → 0.50%), *Butyricicoccus* (0.05% → 0.11%), and *Megasphaera* (0.02% → 0.36%) (*P*<0.05, Fig. 5E). Maternal dietary *L. reuteri* intake did not influence the α-diversity (except for the richness index at day 3) or β-diversity of the fecal microbiota in piglets at later time points (Additional file 1: Fig. S8A-8D; Additional file 2: Table S1h).

Oral administration of *L. reuteri* significantly increased the α-diversity of the fecal microbiota in piglets after weaning (Fig. 5B) and at day 3 (Additional file 1: Fig. S8A). For the overall microbiota composition, oral intake explained 4.5% (*P*=0.016) and 5.3% (*P*=0.006) of the interindividual variation according to PERMANOVA based on Bray–Curtis metrics at day 28 (Fig. 5D) and day 3 (Additional file 2: Table S1h), respectively. Since maternal dietary *L. reuteri* intakes only influenced the microbiota composition of newborn piglets, subsequent analyses of the change in taxa at the genus level focused on the oral administration of *L. reuteri*, and the Wilcoxon rank-sum test was used to analyze the difference. At day 3, oral *L. reuteri* intake significantly decreased the abundances of *Escherichia/Shigella* (35.77% → 27.81%) and *Enterococcus* (16.83% → 10.53%), and increased the abundances of *Clostridium_XlVa* (2.81% → 4.5%), *Clostridium_XlVb* (0.54% → 0.75%), *Lactobacillus* (2.82% → 7.1%), and *Ruminococcus* (0.002% → 0.01%) (*P*<0.1, Fig. 5F). At day 28, the abundances of *Blautia* (3.07% → 1.87%), *Succinivibrio* (2.90% → 1.10%), *Terrisporobacter* (0.76% → 0.11%), and *Clostridium_XlVb* (0.13% → 0.06%) were higher in the Oral group than that in the Nonoral group (Additional file 1: Fig. S8E). OTU-level clustering of the longitudinal samples indicated clear variation in the microbiota profiles between the Oral and Nonoral groups (Additional file 1: Fig. S8F). The clustering of the Oral group included four stages, namely, day 0, day 3–day 7, day 14–day 21, and day 28, while that of the Nonoral groups included five stages.

We also examined the effect of maternal dietary supplementation and oral intake of *L. reuteri* on fecal microbiota maturation in piglets during early life. To identify the age-dependent development of the fecal microbiota in piglets, the random forest model derived from the relative abundance of all species in the control group (neither dietary supplementation nor oral *L. reuteri* intake) was regressed against the chronological age of the piglets at different time points. The top 19 age-specific taxa were identified based on tenfold cross-validation with five repeats. Then, these top taxa in the control group were used to build the random forest model (90.86% variation explained) and employed to define the microbiota age across all four groups (Additional file 1: Fig. S8G). The relative microbiota maturity in the I5007_Oral, Control_Oral, and Control_Nonoral groups was further compared to that in the Control_Nonoral group. The development of the natural fecal microbiota followed a smooth line that showed a gradual increase with age (Additional file 1: Fig. S8H). The development of the microbiota in the other three groups exhibited a smooth curve that featured an early-maturation pattern, especially in the I5007_Oral group. Both maternal dietary supplementation and oral intake influenced the relative maturity of the fecal microbiota in piglets, and the maternal effects were stronger than those of oral intake (Fig. 5G). Specifically, the relative microbiota maturity in the I5007 group was higher at day 0 (*P*<0.01, Wilcoxon rank-sum test) but significantly lower at the later time points (day 14, day 21, and day 28). In addition, compared to the Nonoral group, the Oral group exhibited higher relative microbiota maturity values at day 28 (*P*<0.05), and these values did not change at the other ages.

**Fig. 6:**
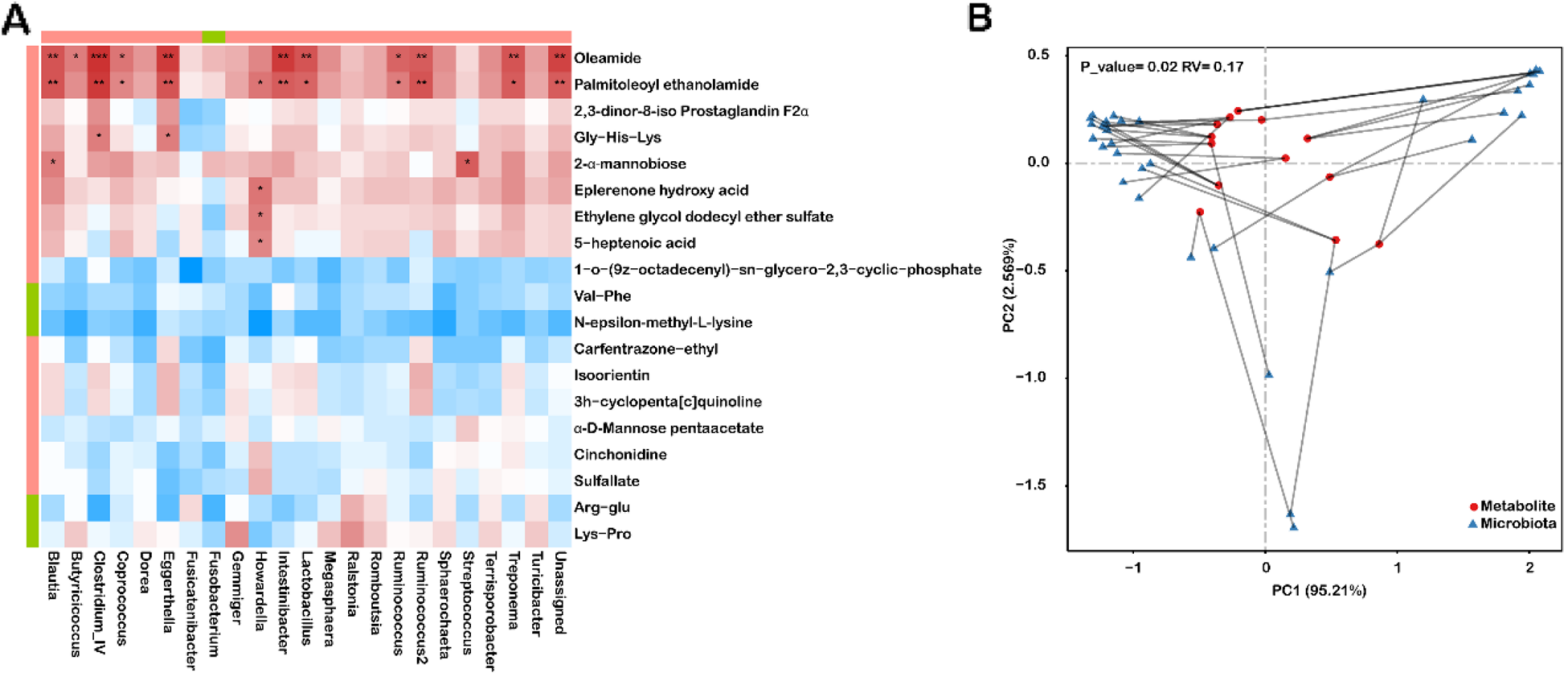
The relationship between serum metabolite features and meconium microbiota. **A** Heatmap of Spearman’s rank correlations between differential metabolites in umbilical cord blood serum and significant genera in the meconium. The correlation coefficients in each square represent positive (red) and negative (blue) correlations. The color intensity is proportional to the absolute value of Spearman’s rank correlation values. Statistically significant correlations are marked with *P <0.05, **P<0.01, or ***P <0.001. The upper panel represents the control-enriched (green) and I5007-enriched (red) general metabolites, and the left panel represents the control-enriched (green) and I5007-enriched (red) metabolites with differential abundance between the two groups. **B** Coinertia analysis of the relationship among 13 differential serum metabolites and 21 differentially abundant genera in the meconium using the Bray–Curtis metric. Blue and red represent microbiota and metabolites, respectively. PCs represent the first 2 principal components from the coinertia analysis. The differences were analyzed by the Monte Carlo test

To explore the potential relationships between the circulating metabolites in cord blood serum and the microbiota composition in the meconium, a correlation matrix was generated for the 19 significantly altered metabolites and 23 differentially abundant genera using Spearman correlation analysis (Fig. 6A). The heatmap revealed that I5007-enriched oleamide and palmitoleoyl ethanolamide were positively correlated with the abundance of several beneficial bacteria, including *Blautia*, *Butyricicoccus*, *Clostridium*_IV, *Lactobacillus*, *Megasphaera*, *Ruminococcus*, and *Ruminococcus2*. A similar result observed with RDA also showed that palmitoleoyl ethanolamide and oleamide significantly contributed to the alterations in microbiota composition (*P* ≤ 0.05) and were positively correlated with the most significant genera (Additional file 1: Fig. S9A). In addition, the levels of 5-heptenoic acid (*R*=−0.59, *P*=0.033) and cinchonidine (*R*=−0.60, *P*=0.029) were negatively correlated with the abundance of *Escherichia/Shigella*, which are a potential pathogen in animals with diarrhea (Additional file 1: Fig. S9C). The CIA also showed that the meconium microbiota and metabolites shared similar trends (Fig. 6B and Additional file 1: Fig. S9B). Notably, the differential metabolites were the main contributors to the correlation between the microbiota and metabolites.

### Maternal dietary supplementation and oral administration of L. reuteri conferred the piglets with growth benefits

**Fig. 7:**
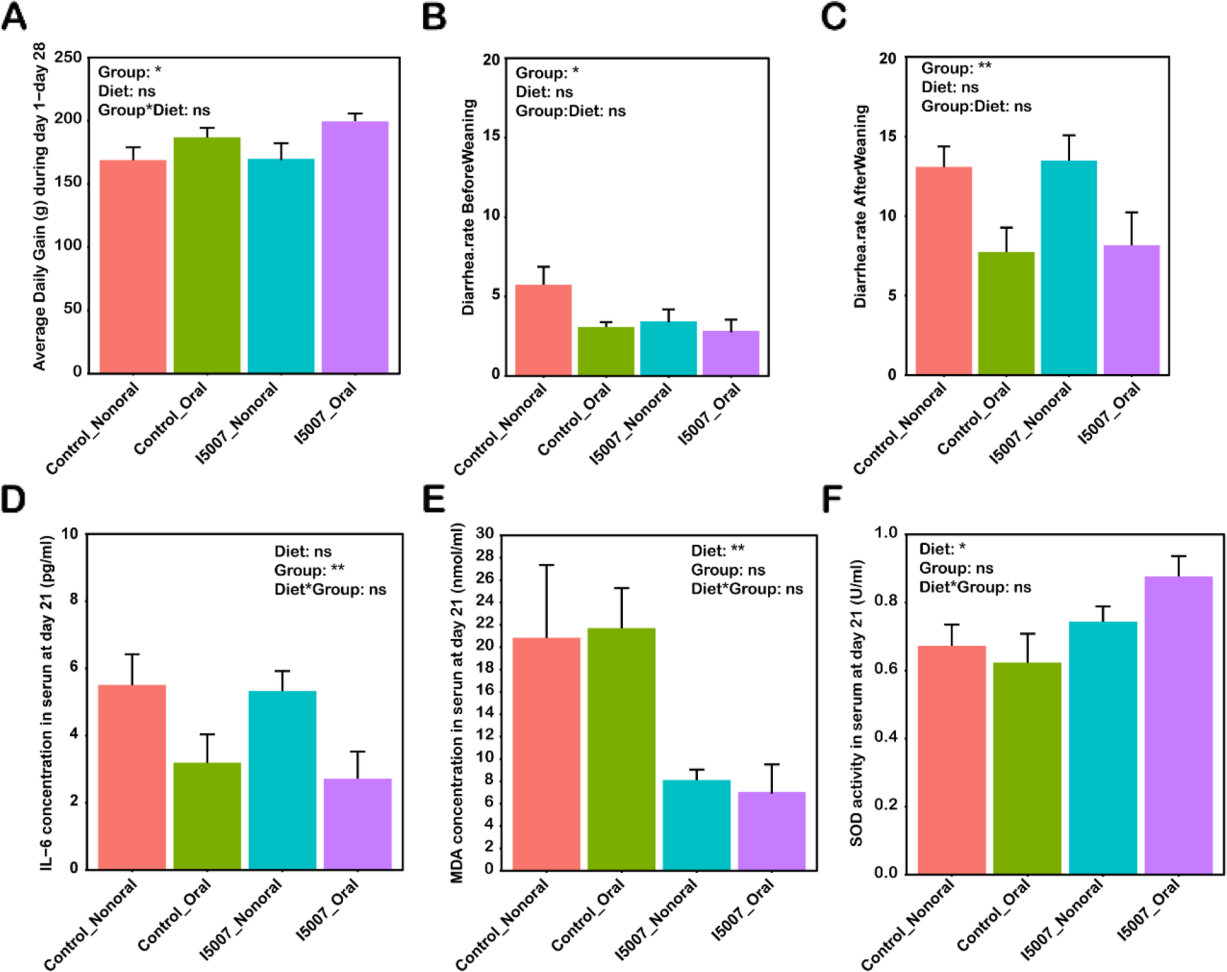
Weight gain, diarrhea incidence, inflammatory cytokines, and antioxidation indices under maternal dietary intake and oral administration of *L. reuteri*. **A** Average daily gain of piglets during the whole trial of 28 days. **B** Diarrhea incidence of piglets during days 1–21 before weaning (**B**) and days 22–28 after weaning (**C**). The levels of the proinflammatory cytokine IL-6 (**D**) and MDA (**E**) in the serum of piglets at day 21. **F** The enzyme activity of SOD in the serum of piglets at day 21. U is the international unit of enzyme activity. The data are shown as the mean ± SEM. Differences among treatments were analyzed by two-way ANOVA. ns, not significant; *P <0.05; **P<0.01. Diet indicates dietary supplementation with *L. reuteri* or not; group indicates the oral administration of *L. reuteri* or not

To evaluate the performance in growth promotion, we measured the average daily gain, weight change, and diarrhea incidence of piglets. During the whole trial of 28 days, oral administration of I5007 significantly increased the average daily gain of piglets, but maternal dietary intake did not influence this parameter (Fig. 7A). Notably, the I5007_Oral group had the highest average daily gain compared with the other three groups. Similarly, oral administration but not dietary intake of I5007 resulted in a higher average daily gain from day 22 to day 28 (Additional file 1: Fig. S10A). Both maternal dietary intake and oral administration of I5007 increased the weight changes on day 21 and day 28, and these two methods in combination performed the best (Additional file 1: Fig. S10B). Diarrhea is a key negative factor that increases mortality and reduces the growth performance of piglets during early life. We next examined the effect of *L. reuteri* on diarrhea incidence in piglets. The results showed that oral administration of I5007 promoted diarrhea resistance during the preweaning and postweaning phases (Fig. 7B, C). We also found a significant reduction in the levels of the proinflammatory cytokine IL-6 under oral administration of I5007 in the serum of piglets at day 21 (Fig. 7D). Interestingly, there was no effect of oral administration of I5007 on the activity of SOD and the concentration of MDA, but maternal dietary intake significantly increased the SOD activity and decreased the MDA level in the serum of piglets on day 21 (Fig. 7E, F). We assumed that I5007 treatment also changed the levels of proinflammatory cytokines and MDA, as well as SOD activity, in the cord blood serum of sows. The results showed that dietary I5007 intake only decreased the concentrations of the proinflammatory cytokines TNF-α and IL-6 in the cord blood serum (Additional file 1: Fig. S11A and S11B).

### Dietary supplementation and oral administration of L. reuteri modulated diarrhea-associated bacteria

Since diarrhea alters the fecal microbiota composition (Additional file 1: Fig. S12), we further determined whether the dietary supplementation and oral administration of *L. reuteri* could influence the structures of key microbes associated with diarrhea in piglets during early life. We first built a machine learning model. Using the mRMR method, the top 19 OTUs had the highest MCC values and were chosen as the final markers to construct the SVM classifier (Additional file 1: Fig. S13A). These OTUs in the model were primarily members of Ruminococcaceae, Lachnospiraceae, and Enterococcaceae (Additional file 2: Table S1i). The predictive power of this model was evaluated by the area under the curve (AUC) of the receiver operating characteristic (ROC). This SVM classifier showed a high AUC of 97% and a 95% confidence interval of 93.4 to 100% for the independent test data set (Additional file 1: Fig. S13B). To further evaluate whether the classifier could be generalized for prediction in other samples, we used an independent validation cohort and found a moderately good performance (AUC = 73.4%), although not as good as that in the original test cohort (Additional file 1: Fig. S13B). Next, we examined the effects of maternal dietary intake and oral administration of *L. reuteri* on the alteration in α-diversity and β-diversity within these 19 OTUs (Additional file 1: Fig. S14). The PCoA showed that dietary intake of *L. reuteri* regulated the overall microbial structure based on the Bray–Curtis distance and explained 4.4% of the interindividual variation at day 3. Moreover, the richness, Shannon and Simpson indices revealed a change in the richness and diversity of these OTUs after 28 days of oral administration of *L. reuteri*.

## Discussion

In the present study, we used a combination of taxonomic, metagenomic, and metabolomic approaches, and the results revealed that *L. reuteri*, administered either directly or indirectly through vertical transfer, improved the development and maturation of fecal microbiota and subsequently promoted the early growth of piglets.

These results are consistent with a previous report showing that *L. reuteri* had little effect on the fecal microbiota composition in healthy adults [70]. The administration of *L. reuteri* to sows in the diet for 4 weeks did not change their fecal microbiota composition, which may be explained by the fact that the microbiomes of adult individuals are very mature and stable, making it difficult for exogenous probiotics to colonize the intestine in large numbers and affect the community. Although whether the microbial colonization of newborns begins prior to birth remains controversial, it is clear that diverse microbes from the maternal gut bacteria can be vertically transmitted to the infant gut and might serve as seeding bacteria to prime the development of the infant gut microbiome at subsequent time points [19, 71]. Remarkably, the taxonomic composition and functional profiles of the colostrum were influenced by maternal dietary supplementation with *L. reuteri*, as the biodiversity and microbial interactions in these colostrum samples were significantly different from those of normal colostrum microbiota. The bacteria belonging to the genera *Brachybacterium*, which was identified as the commensal microorganism in the milk microbiome, were significantly enriched in the colostrum of *L. reuteri* treated group. *Brachybacterium* has the ability to rapidly convert lactose to lactate [72], which is consistent with our result that the concentration of lactate was higher in the colostrum of *L. reuteri* treated groups. Species network analysis of colostrum of *L. reuteri* treated group and normal colostrum showed that the former had a more complex microbial community. Generally, microbiota with enhanced biodiversity are more resilient and adaptable to stress and less susceptible to pathogen infection than those with less diversity [73]. The “core” species with more than 10 nodes in the *L. reuteri* treated group network were positively associated with enriched pathways, including glycolysis/gluconeogenesis and lysine biosynthesis. Moreover, a functional KEGG pathway, bacterial invasion of epithelial cells, was enriched and significantly associated with the enriched species in the normal colostrum. Taken together, our results indicated that maternal dietary supplementation with *L. reuteri* could markedly modulate the microbiota structure and functional composition in the colostrum.

Notably, maternal diet supplementation with *L. reuteri* had a strong overall impact on the fecal microbiota composition in newborn piglets. Relative to those in the control group, most of the bacteria that differentially colonized the intestines of newborn piglets have been reported to have particularly important effects on improving host health during neonatal life (e.g., *Lactobacillus*, *Blautia*, *Ruminococcus*, and *Clostridium_IV* ) [74]. Moreover, the oral administration of *L. reuteri* also increased the abundances of *Lactobacillus*, *Eubacterium*, and *Ruminococcus* and greatly decreased the abundance of the *Escherichia/Shigella*. *Escherichia coli* is a keystone diarrhea-causing enteropathogen during the suckling period and has a severe impact on animal health [75]. This result indicated that *L. reuteri* could inhibit the colonization of the pathogen in the piglet intestine indirectly or directly and thus decrease the incidence of diarrhea before weaning. Interestingly, neither maternal dietary supplementation nor oral administration affected the fecal microbiota composition during the middle (days 7 and 14) and late (day 21) stages of nursing. This result may be explained by the fact that the development and maturation of the gut microbiota during early life is a dynamic and nonrandom process that is influenced by various factors, such as the mode of delivery [13], host genetics [76], feeding mode [77], and antibiotic usage [78]. The “natural” developmental characteristics of the infant microbiome may overshadow the effect of *L. reuteri* supplementation. Our findings confirmed that the oral administration of *L. reuteri* reproduced the modulation of the fecal microbiota composition and reduced diarrhea after weaning (day 28) despite the cessation of maternal *L. reuteri* administration. The relative abundances of *Blautia* and *Butyricicoccus* were higher in the probiotic group at birth and after weaning than those in the control group. *Blautia* and *Butyricicoccus* are common butyrate-producing bacteria in the intestine [74, 79]. The regulatory effect of butyrate on fecal immunity and gut health has been extensively studied and recognized [80–82]. These findings were consistent with previous studies showing that dietary supplementation with *L. reuteri* increased the concentration of SCFAs in piglet feces [83, 84], suggesting *L. reuteri* drives the enrichment of several functional microbes to modulate the development and maturation of the fecal microbiota in piglets.

Metabolic profiling revealed significant pattern differences induced by the maternal dietary supplementation with *L. reuteri.* Among the significantly changed metabolites, oleamide and palmitoleoyl ethanolamide were positively correlated with the most enriched genus in the *L. reuteri* group. This result suggested that these two metabolites may drive the alteration of fecal microbiota between the two groups in newborn piglets. Previous reports have shown that oleamide and palmitoleoyl ethanolamide have anti-inflammatory effects in vitro [85] and in animal models [86]. We also observed that the serum concentrations of the proinflammatory factors TNF-α and IL6 were decreased after the consumption of *L. reuteri.* Consistent with our study, supplementation with other probiotics, namely, *Pediococcus pentosaceus* KID7 and *Lactobacillus rhamnosus* LRa05, increased the levels of oleamide [87] and palmitoleoyl ethanolamide [88], respectively. One study reported that oleamide was found in the supernatant of another *Lactobacillus* species, *Lactobacillus sakei* probio65 [89]. In addition, we observed that the differential metabolites cinchonidine and 5-heptenoic acid were negatively correlated with the abundance of *Escherichia*/*Shigella*. The former was reported to have antibacterial activity [90], and the latter was shown to attenuate bacterial LPS-induced inflammation [91]. However, whether *L. reuteri* modulate these key metabolites directly or indirectly via the metabolism of the host remains unclear, and the mechanism requires further investigation. In addition, coinertia analysis showed marked covariation between serum metabolites and the fecal microbiota in newborn piglets. This strongly suggested that the microbiota of newborn piglets was modulated by metabolites during mother-fetal nutrition transfer via the umbilical cord blood. In summary, our data suggest that the structural characteristics of the fecal microbiota in newborn piglets were associated with the alteration of metabolite profiles in umbilical cord blood serum caused by supplementation with *L. reuteri* in the maternal diet.

The development and maturation of microbiota in early life was shown to affected immune response, to promote growth and to potentially decrease disease susceptibility [2]. Diarrhea is a key negative factor that increases mortality and reduces the growth performances of piglets during early life. Our results suggested that the oral administration of I5007 promoted diarrhea resistance and decreased inflammation. Consistent with previous studies, the addition of *Lactobacillus* strains promoted growth and immunological parameters in broilers [78] and piglets [83, 92]. Maternal supplementation of *L. reuteri* enhanced the antioxidant ability of offspring. This result may be explained by the fact that *L. reuteri* modulated several key microbes in the piglets. The maternal supplementation of *L. reuteri* was confirmed to affect the fecal microbiota richness of piglets on day 3, which was potentially due to the regulation of *L. reuteri* on colostrum. Notably, both the maternal dietary intake and oral administration of I5007 increased the weight changes in piglets, and these two methods exhibited the best weight changes.

Consistent with a previous study, diarrhea disrupted the intestinal microbiota, characterized by the presence of distinctive microbial markers belonging to *Enterococcus* spp. and *Prevotella* spp. [93]. Among the 19 optimal OTU-based microbial markers, several species have been reported to impact the host functionally or pathogenically. Clostridium species belonging to Clostridium cluster IV promote the development of the intestinal immune system [94]. The genus *Facklamia* was reported to be enriched upon the administration of *L. rhamnosus* GG to pigs challenged with *Salmonella enterica* [95]. Moreover, we observed that the maternal dietary supplementation and oral administration of *L. reuteri* regulated the changes in the diversity of these microbial biomarkers associated with diarrhea. However, we observed that the predictive accuracy was decreased in the independent validation cohort, which may have been due to the high intercohort variability. Thus, these results revealed that *L. reuteri* intake by both sows and piglets promoted growth and diarrhea resistance, possibly by driving the dynamics of key fecal microorganisms in piglets during early life.

Indeed, a small number of studies have provided preliminary evidence that the maternal dietary supplementation or oral administration of *L. reuteri* modulates the infant microbiota [13, 27, 29, 96]. These studies focused mainly on alterations in the gut microbiome and did not examine maternal changes other than those in the intestinal microbiota. Our study furthers our global understanding of the functional variations in the metabolite features of umbilical cord blood serum and in the metabolome profiles in colostrum, as well as in infant intestinal microbiota, as determined by multiomics analysis. In addition, the modulation of diarrhea-associated microbial markers by *L. reuteri* further illustrates that probiotics are an effective method for preventing diarrhea in piglets during early life, especially given that the use of antibiotics is gradually being prohibited. However, future studies are needed to (1) investigate the mechanism by which key metabolites modulate the microbiota composition in the intestines of infants as well as how they impact the intestinal immune system during the neonatal period and (2) examine the potential interactions among the metabolites, key microbes, and *L. reuteri* during mother-infant transmission.

## Conclusions

In conclusion, both maternal dietary supplementation and oral administration of *L. reuteri* modulate and accelerate the maturation of the fecal microbiota composition in piglets during early life. The beneficial effects of *L. reuteri* may improve through the regulation of metabolite profiles in the blood of sow during pregnancy and microbes in the colostrum of sows, leading to manipulation of the infant fecal microbiota and immune function. Thus, our findings support the utility of probiotics as an effective means to improve the dysfunctional microbiota and potentially promote the development of the infant fecal microbiome and gut health during the early postnatal period of life.

## Supplementary Information

**Additional file 1: Figure S1**. The effects of *L*. *reuteri* supplementation on the microbiota composition in the intestine of sows. (A) Dynamic changes in α-diversity (richness, Shannon and Simpson index) between two groups across different ages. The data are expressed as the mean ±SEM. Differences were analyzed by two-way ANOVA based on the Scheirer-Ray-Hare test. (B) PCoA plot based on the Bray–Curtis distance of microbiota composition. Significance was calculated using PERMANOVA. (C) Bray–Curtis dissimilarity between the *L. reuteri* I5007 and control groups across different ages. The median of the data is shown. Differences were analyzed by two-way ANOVA based on the Scheirer-Ray-Hare test. (D) Histogram showing the relative abundances of the five most abundant phyla in the intestine of sows over time. (E) Heatmap showing the genera that were significantly affected by supplementation with *L. reuteri* I5007 on a certain day. The data are expressed as the mean relative abundance (%) in each group. The Wilcoxon rank-sum test was used to analyze variation between two groups at the same time point. Diet indicates dietary supplementation with *L. reuteri* or not; group indicates the oral administration of *L. reuteri* or not. **Figure S2**. Alterations in the colostrum microbial composition. Histogram of the structural composition of the microbiota at the phylum level (top 6) (A) and the family level (top 10) (B) in the colostrum samples. Significance was measured using the Wilcoxon rank-sum test. The horizontal bars within the box represent the median. (C) The α-diversity of bacteria was measured using the richness, Shannon and Simpson indices. (D) PCoA plots of significantly different species based on Bray–Curtis distance. **Figure S3**. Significantly different species between the I5007 and control groups. Histograms of significantly altered species with the criteria LDA>2 and P<0.05 from metagenome data between the two groups. The color of the bar indicates enrichment of the species in the I5007 (green) and control (red) groups. **Figure S4**. Correlation analysis of KEGG pathways and species in the colostrum. Heatmap of Spearman’s rank correlations between differential KEGG pathways and species from the colostrum metagenome data. Correlation coefficients in each square represent positive (red) and negative (blue) correlations. The color intensity is proportional to the absolute values of Spearman’s rank correlation. Statistically significant correlations are marked with *P <0.05, **P<0.01, and ***P <0.001. The left panel represents the control-enriched (green) and I5007-enriched (red) metabolites or KEGG pathways between the two groups. **Figure S5**. The microbial gene functions annotated in CAZy, the concentration of acids and the relationship between the serum metabolites and species in colostrum. (A) PCoA based on the Bray–Curtis distances of CAZy orthologs between the I5007 and control groups. Boxplots showing the concentrations of lactate (B) and propionate (C) between the I5007 and control groups at different time points. Diet means dietary supplementation of *L. reuteri* or not; Group means oral administration of *L. reuteri* or not. **Figure S6**. WGCNA analysis of the metabolites features in the umbilical cord blood serum. Box plots of eigenmetabolite levels of the MEdarkgray, MEpink, MElightcyan and MEblack modules between the I5007 and control groups. The Wilcoxon rank-sum test was used to analyze the variation between two groups. **Figure S7**. Multiomic association studies on the microbiota of sows and the serum metabolome. (A) Coinertia analysis of the relationships between all serum metabolites and 112 differential species in colostrum. Coinertia analysis of the fecal microbiota in sows and the significantly different (B) and all (C) serum metabolites. PCs represent the two first principal components from the CIA. **Figure S8**. Effect of *L. reuteri* on the fecal microbiota in piglets of different ages. Boxplots of α-diversity (richness, Shannon and Simpson index) on (A) day 3, (B) day 7, (C) day 14, and (D) day 21. The median of the data is shown. Differences were analyzed by two-way ANOVA based on the Scheirer-Ray-Hare test at the same time point. (E) Bar graph showing significant differentially abundant genera between the Oral and Nonoral groups at day 28. The data are expressed as the average relative abundance of genera in each group. The Wilcoxon rank-sum test was used to analyze variation between two groups. (F) Heatmap showing the clustering of the fecal microbiota at the OTU level in the Oral and Nonoral groups. (G) The top 19 age-specific taxa identified by applying random forest regression of their relative abundances. These biomarker taxa are ranked in descending order of importance to the accuracy of the model. The inset of (G) shows the tenfold cross-validation error as a function of the number of input taxa used to regress against the age of piglets and in the order of variable importance. (H) Microbiota age in different groups predicted by the random forest regression model. The curve is a smoothed spline fit between microbiota age and chronological age, and each circle represents an individual sample. Diet indicates dietary supplementation with *L. reuteri* or not; group indicates the oral administration of *L. reuteri* or not. **Figure S9**. Alterations in serum metabolite features and the relationship with the meconium fecal microbiota. (A) Redundancy analysis (RDA) plot was generated with differential metabolites significantly correlated with at least one differentially abundant genus. Each black circle indicates all the serum samples. Blue arrows indicate the differential serum metabolites (dependent variables). Red arrows indicate the differentially abundant genera (independent variable). The relationship of each independent variable with another variable is equal to the cosine of the angle between the arrows; a small angle indicates a high positive correlation, and opposite directions indicate a negative correlation. (B) Coinertia analysis of the relationship between all serum metabolites and 23 differentially abundant genera in the meconium using the Bray–Curtis metric. Blue and red represent the microbiota and metabolites, respectively. (C) The scatter plots show the correlation of the level of 5-heptenoic acid and cinchonidine (log10 conversion) with the relative abundance of *Escherichia/Shigella*. **Figure S10**. Effects of maternal dietary intake and oral administration of *L. reuteri* on the growth performance of piglets. (A) Average daily weight gain of piglets from day 22 to day 28; (B) Weight change of piglets during day 1-day 28 under maternal dietary intake and oral administration of *L. reuteri*. The data are expressed as the mean ± SEM. The data are shown as the mean ± SEM. Differences among groups were analyzed by two-way ANOVA. ns, not significant; *P <0.05; **P<0.01. Diet means dietary supplementation of *L. reuteri* or not; group means oral administration of *L. reuteri* or not. **Figure S11**. Concentration of proinflammatory cytokines in the cord blood of sows. The bars show the levels of TNF-α (A) and IL-6 (B) between the I5007 and control groups. The data are shown as the mean ± SEM. Differences between the control and I5007 groups were analyzed by unpaired t-tests (two-tailed). *P <0.05. IL-6, interleukin 6; TNF-α, tumor necrosis factor-α. **Figure S12**. The overall microbiota composition between diarrheic and healthy individuals. Principal coordinates analysis (PCoA) plots of all OTUs based on Bray–Curtis distance from day 3 to day 28. Red points represent the samples of individuals with diarrhea, and green points represent the samples of healthy individuals. **Figure S13**. OTU-based classifier used to distinguish diarrheic and healthy individuals. (A) The mRMR method was used to identify diarrhea-associated biomarkers. The IFS curve of the top 100 mRMR OTUs. The x-axis shows the number of OTUs, and the y-axis shows the prediction performance. The peak MCC value was 0.832 when 19 OTUs were chosen. (B) Receiver operating characteristic (ROC) curves for the diarrheic and healthy individuals; 95% confidence intervals (CIs) are indicated by error bars. The blue line represents the ROC curve of the test data set, and yellow represents the ROC curve of the independent validation data set. **Figure S14**. Alteration in α-diversity and β-diversity within 19 OTUs. (A) PCoA plots of 19 OTUs based on Bray–Curtis distance at day 3. (B) Boxplots of α-diversity (richness, Shannon and Simpson index) at day 28. The median of the data is shown. Differences were analyzed by two-way ANOVA based on the Scheirer-Ray-Hare test. ns, not significant; *P <0.05; **P<0.01. Diet means dietary supplementation of *L. reuteri* or not; group means oral administration of *L. reuteri* or not.

**Additional file 2: Table S1a**. The relative abudance of taxa at phylum and family level in the feces and colostrum. **Table S1b**. Description of 112 differential species between I5007 and control groups. **Table S1c**. Global network parameters of 112 differential species. **Table S1d**. Description of taxa information for each node in I5007 network. **Table S1e**. Description of taxa information for each node in control network. **Table S1f**. Description of differential KOs between I5007 and control groups. **Table S1g**. Description of metabolite modules of serum metabolites under polar ionic mode. **Table S1h**. PERMANOVA of fecal microbiota in piglets at different age. **Table S1i**. Description of taxa information for 19 microbial biomarkers.

## Data Availability

The raw Illumina sequence data of sows and piglets and the independent validation cohort generated in the present study are available in the sequence read archive (SRA) at NCBI under Bioproject accessions # PRJNA701781 and # PRJNA730972, respectively. The metagenome sequence data of the colostrum are available in NCBI SRA under Bioproject accession #PRJNA701802. All of these data sets will be released on 31 December 2023.

## Abbreviations

CFU: Colony-forming unit
PCoA: Principal coordinate analysis
KEGG: Kyoto Encyclopedia of Genes and Genome
OPLS-DA: Orthogonal projection to latent structures discriminant analysis
CIA: Coinertia analysis
WGCNA: Weighted correlation network analysis
SCFAs: Short-chain fatty acids
LDA: Linear discriminant analysis
LEfSe: Linear discriminant analysis effect size
RV: Regression vector
ADG: Average daily gain
TNF-α: Tumor necrosis factor
IL-6: Interleukin 6
MDA: Malondialdehyde
SOD: Superoxide dismutase
mRMR: Minimal redundancy maximal relevance
LOOCV: Leave-one-out cross-validation
MCC: Matthews correlation coefficient
ROC: Receiver operating characteristic
PERMANOVA: Permutational multivariate analysis of variance
RDA: Redundancy analysis
FDR: False discovery rate
BH: Benjamini-Hochberg
SEM: Standard error of the mean
