# Supplementary figures and images for: Lactobacillus reuteri improves the development and maturation of fecal microbiota in piglets through mother-to-infant microbe and metabolite vertical transmission
Source: Microbiome. 2022 Dec 2;10:211. doi: 10.1186/s40168-022-01336-6 (PMC9717520; doi:10.1186/s40168-022-01336-6)

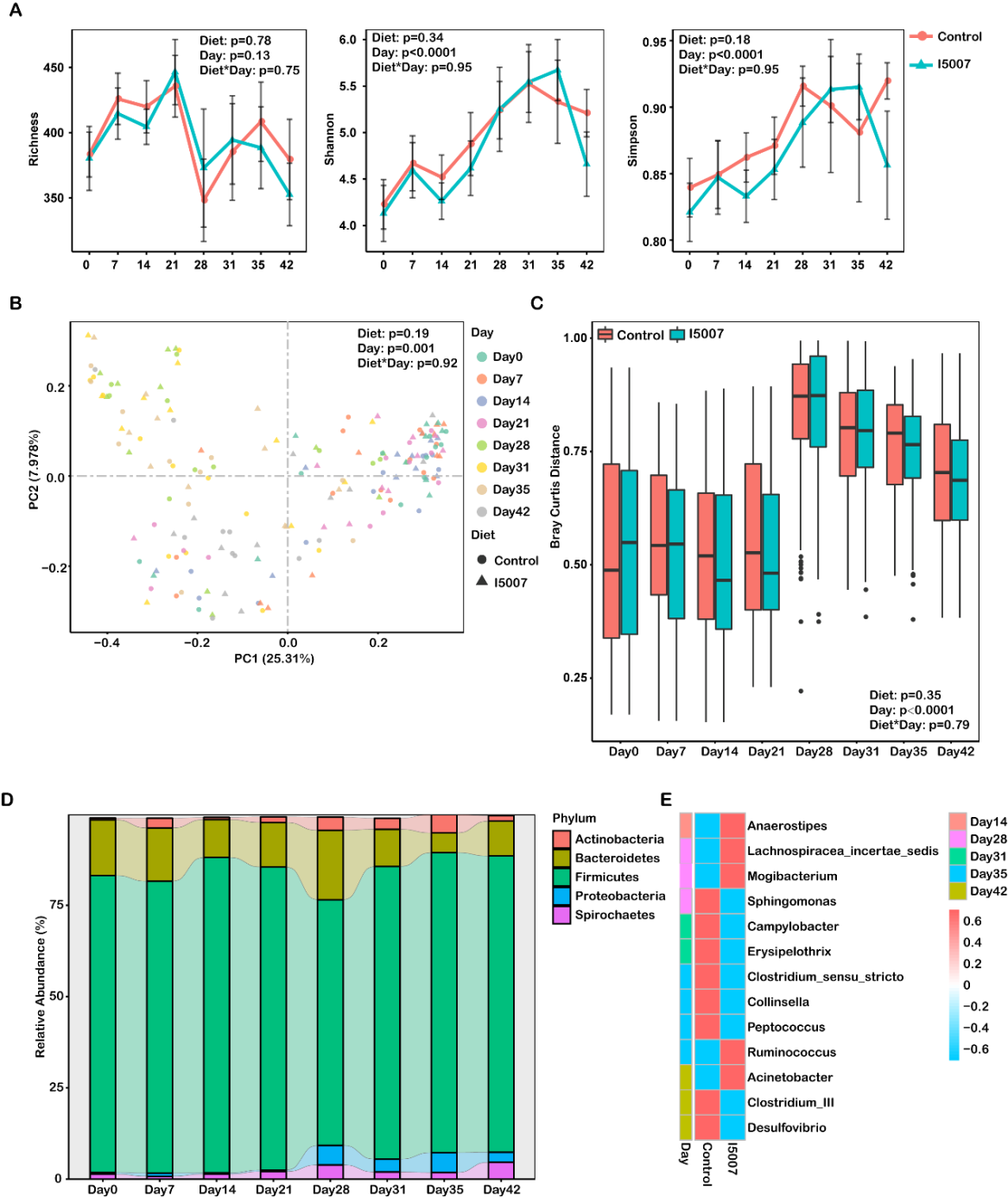

Supplement: Supplementary file 2 — Additional file 1: Figure S1. The effects of L. reuteri supplementation on the microbiota composition in the intestine of sows. (A) Dynamic changes in α-diversity (richness, Shannon and Simpson index) between two groups across different ages. The data are expressed as the mean ±SEM. Differences were analyzed by two-way ANOVA based on the Scheirer-Ray-Hare test. (B) PCoA plot based on the Bray–Curtis distance of microbiota composition. Significance was calculated using PERMANOVA. (C) Bray–Curtis dissimilarity between the L. reuteri I5007 and control groups across different ages. The median of the data is shown. Differences were analyzed by two-way ANOVA based on the Scheirer-Ray-Hare test. (D) Histogram showing the relative abundances of the five most abundant phyla in the intestine of sows over time. (E) Heatmap showing the genera that were significantly affected by supplementation with L. reuteri I5007 on a certain day. The data are expressed as the mean relative abundance (%) in each group. The Wilcoxon rank-sum test was used to analyze variation between two groups at the same time point. Diet indicates dietary supplementation with L. reuteri or not; group indicates the oral administration of L. reuteri or not. Figure S2. Alterations in the colostrum microbial composition. Histogram of the structural composition of the microbiota at the phylum level (top 6) (A) and the family level (top 10) (B) in the colostrum samples. Significance was measured using the Wilcoxon rank-sum test. The horizontal bars within the box represent the median. (C) The α-diversity of bacteria was measured using the richness, Shannon and Simpson indices. (D) PCoA plots of significantly different species based on Bray–Curtis distance. Figure S3. Significantly different species between the I5007 and control groups. Histograms of significantly altered species with the criteria LDA>2 and P<0.05 from metagenome data between the two groups. The color of the bar indicates enrichment of the species [file 40168_2022_1336_MOESM1_ESM.zip › Figure S1.docx]

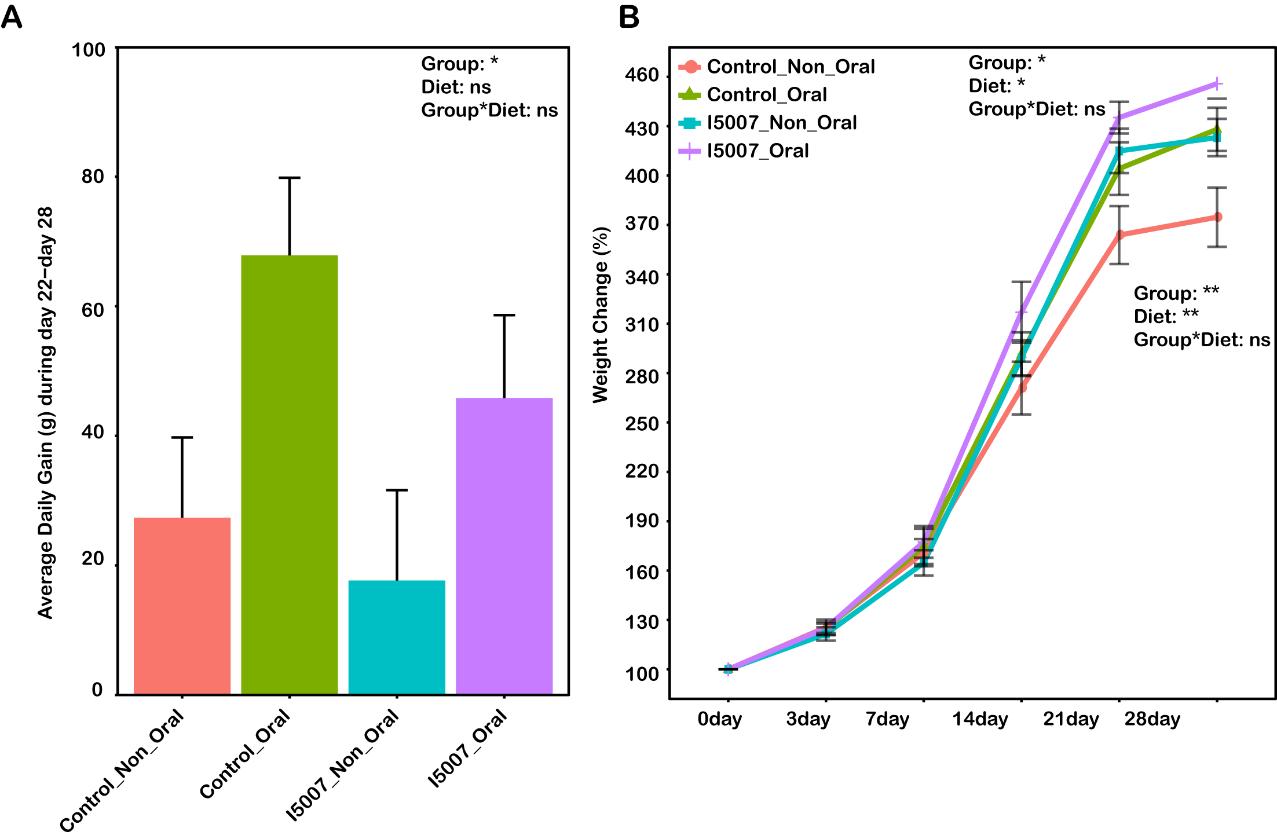

Supplement: Supplementary file 2 — Additional file 1: Figure S1. The effects of L. reuteri supplementation on the microbiota composition in the intestine of sows. (A) Dynamic changes in α-diversity (richness, Shannon and Simpson index) between two groups across different ages. The data are expressed as the mean ±SEM. Differences were analyzed by two-way ANOVA based on the Scheirer-Ray-Hare test. (B) PCoA plot based on the Bray–Curtis distance of microbiota composition. Significance was calculated using PERMANOVA. (C) Bray–Curtis dissimilarity between the L. reuteri I5007 and control groups across different ages. The median of the data is shown. Differences were analyzed by two-way ANOVA based on the Scheirer-Ray-Hare test. (D) Histogram showing the relative abundances of the five most abundant phyla in the intestine of sows over time. (E) Heatmap showing the genera that were significantly affected by supplementation with L. reuteri I5007 on a certain day. The data are expressed as the mean relative abundance (%) in each group. The Wilcoxon rank-sum test was used to analyze variation between two groups at the same time point. Diet indicates dietary supplementation with L. reuteri or not; group indicates the oral administration of L. reuteri or not. Figure S2. Alterations in the colostrum microbial composition. Histogram of the structural composition of the microbiota at the phylum level (top 6) (A) and the family level (top 10) (B) in the colostrum samples. Significance was measured using the Wilcoxon rank-sum test. The horizontal bars within the box represent the median. (C) The α-diversity of bacteria was measured using the richness, Shannon and Simpson indices. (D) PCoA plots of significantly different species based on Bray–Curtis distance. Figure S3. Significantly different species between the I5007 and control groups. Histograms of significantly altered species with the criteria LDA>2 and P<0.05 from metagenome data between the two groups. The color of the bar indicates enrichment of the species [file 40168_2022_1336_MOESM1_ESM.zip › Figure S10.docx]

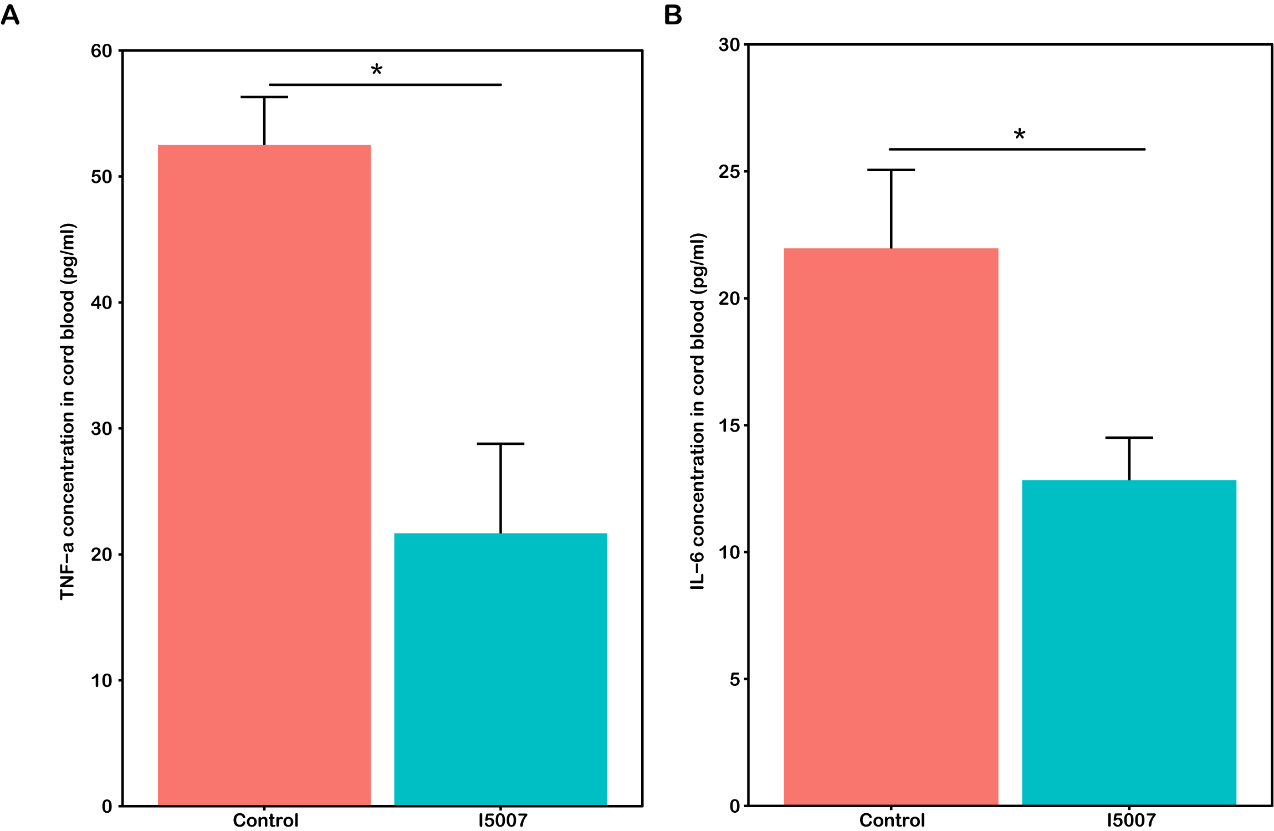

Supplement: Supplementary file 2 — Additional file 1: Figure S1. The effects of L. reuteri supplementation on the microbiota composition in the intestine of sows. (A) Dynamic changes in α-diversity (richness, Shannon and Simpson index) between two groups across different ages. The data are expressed as the mean ±SEM. Differences were analyzed by two-way ANOVA based on the Scheirer-Ray-Hare test. (B) PCoA plot based on the Bray–Curtis distance of microbiota composition. Significance was calculated using PERMANOVA. (C) Bray–Curtis dissimilarity between the L. reuteri I5007 and control groups across different ages. The median of the data is shown. Differences were analyzed by two-way ANOVA based on the Scheirer-Ray-Hare test. (D) Histogram showing the relative abundances of the five most abundant phyla in the intestine of sows over time. (E) Heatmap showing the genera that were significantly affected by supplementation with L. reuteri I5007 on a certain day. The data are expressed as the mean relative abundance (%) in each group. The Wilcoxon rank-sum test was used to analyze variation between two groups at the same time point. Diet indicates dietary supplementation with L. reuteri or not; group indicates the oral administration of L. reuteri or not. Figure S2. Alterations in the colostrum microbial composition. Histogram of the structural composition of the microbiota at the phylum level (top 6) (A) and the family level (top 10) (B) in the colostrum samples. Significance was measured using the Wilcoxon rank-sum test. The horizontal bars within the box represent the median. (C) The α-diversity of bacteria was measured using the richness, Shannon and Simpson indices. (D) PCoA plots of significantly different species based on Bray–Curtis distance. Figure S3. Significantly different species between the I5007 and control groups. Histograms of significantly altered species with the criteria LDA>2 and P<0.05 from metagenome data between the two groups. The color of the bar indicates enrichment of the species [file 40168_2022_1336_MOESM1_ESM.zip › Figure S11.docx]

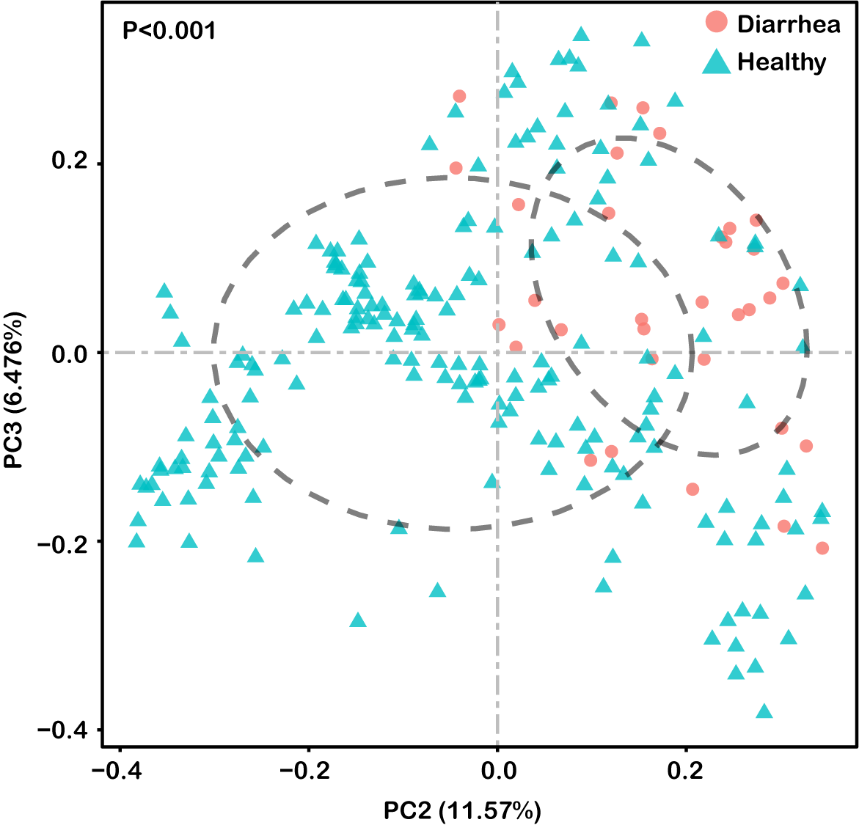

Supplement: Supplementary file 2 — Additional file 1: Figure S1. The effects of L. reuteri supplementation on the microbiota composition in the intestine of sows. (A) Dynamic changes in α-diversity (richness, Shannon and Simpson index) between two groups across different ages. The data are expressed as the mean ±SEM. Differences were analyzed by two-way ANOVA based on the Scheirer-Ray-Hare test. (B) PCoA plot based on the Bray–Curtis distance of microbiota composition. Significance was calculated using PERMANOVA. (C) Bray–Curtis dissimilarity between the L. reuteri I5007 and control groups across different ages. The median of the data is shown. Differences were analyzed by two-way ANOVA based on the Scheirer-Ray-Hare test. (D) Histogram showing the relative abundances of the five most abundant phyla in the intestine of sows over time. (E) Heatmap showing the genera that were significantly affected by supplementation with L. reuteri I5007 on a certain day. The data are expressed as the mean relative abundance (%) in each group. The Wilcoxon rank-sum test was used to analyze variation between two groups at the same time point. Diet indicates dietary supplementation with L. reuteri or not; group indicates the oral administration of L. reuteri or not. Figure S2. Alterations in the colostrum microbial composition. Histogram of the structural composition of the microbiota at the phylum level (top 6) (A) and the family level (top 10) (B) in the colostrum samples. Significance was measured using the Wilcoxon rank-sum test. The horizontal bars within the box represent the median. (C) The α-diversity of bacteria was measured using the richness, Shannon and Simpson indices. (D) PCoA plots of significantly different species based on Bray–Curtis distance. Figure S3. Significantly different species between the I5007 and control groups. Histograms of significantly altered species with the criteria LDA>2 and P<0.05 from metagenome data between the two groups. The color of the bar indicates enrichment of the species [file 40168_2022_1336_MOESM1_ESM.zip › Figure S12.docx]

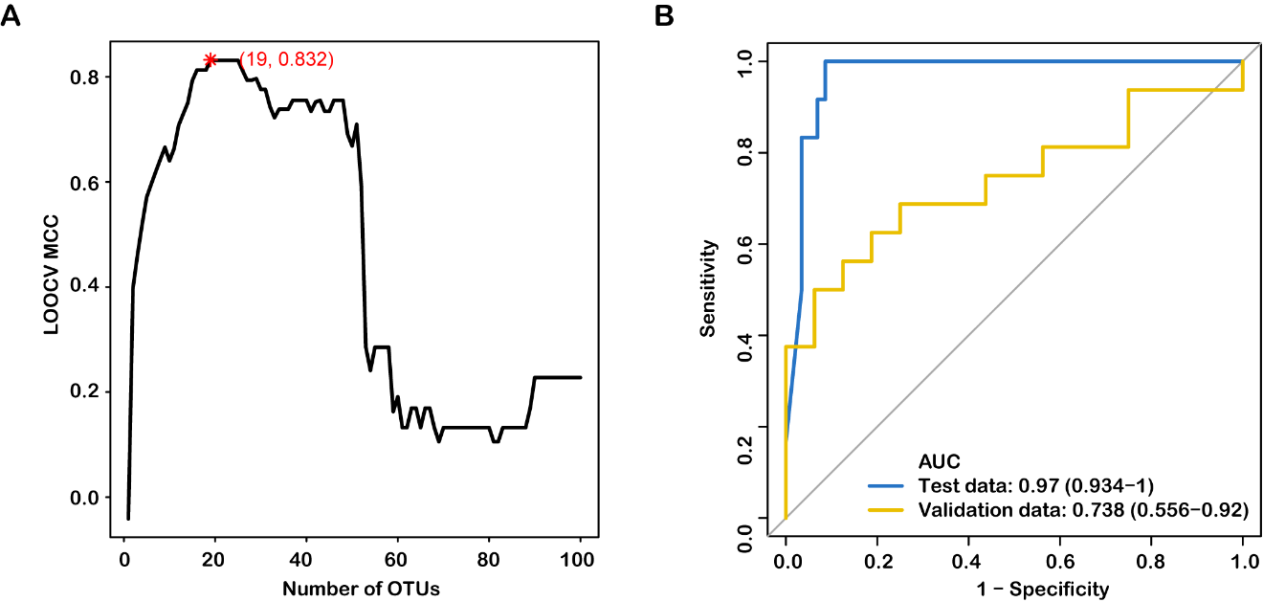

Supplement: Supplementary file 2 — Additional file 1: Figure S1. The effects of L. reuteri supplementation on the microbiota composition in the intestine of sows. (A) Dynamic changes in α-diversity (richness, Shannon and Simpson index) between two groups across different ages. The data are expressed as the mean ±SEM. Differences were analyzed by two-way ANOVA based on the Scheirer-Ray-Hare test. (B) PCoA plot based on the Bray–Curtis distance of microbiota composition. Significance was calculated using PERMANOVA. (C) Bray–Curtis dissimilarity between the L. reuteri I5007 and control groups across different ages. The median of the data is shown. Differences were analyzed by two-way ANOVA based on the Scheirer-Ray-Hare test. (D) Histogram showing the relative abundances of the five most abundant phyla in the intestine of sows over time. (E) Heatmap showing the genera that were significantly affected by supplementation with L. reuteri I5007 on a certain day. The data are expressed as the mean relative abundance (%) in each group. The Wilcoxon rank-sum test was used to analyze variation between two groups at the same time point. Diet indicates dietary supplementation with L. reuteri or not; group indicates the oral administration of L. reuteri or not. Figure S2. Alterations in the colostrum microbial composition. Histogram of the structural composition of the microbiota at the phylum level (top 6) (A) and the family level (top 10) (B) in the colostrum samples. Significance was measured using the Wilcoxon rank-sum test. The horizontal bars within the box represent the median. (C) The α-diversity of bacteria was measured using the richness, Shannon and Simpson indices. (D) PCoA plots of significantly different species based on Bray–Curtis distance. Figure S3. Significantly different species between the I5007 and control groups. Histograms of significantly altered species with the criteria LDA>2 and P<0.05 from metagenome data between the two groups. The color of the bar indicates enrichment of the species [file 40168_2022_1336_MOESM1_ESM.zip › Figure S13.docx]

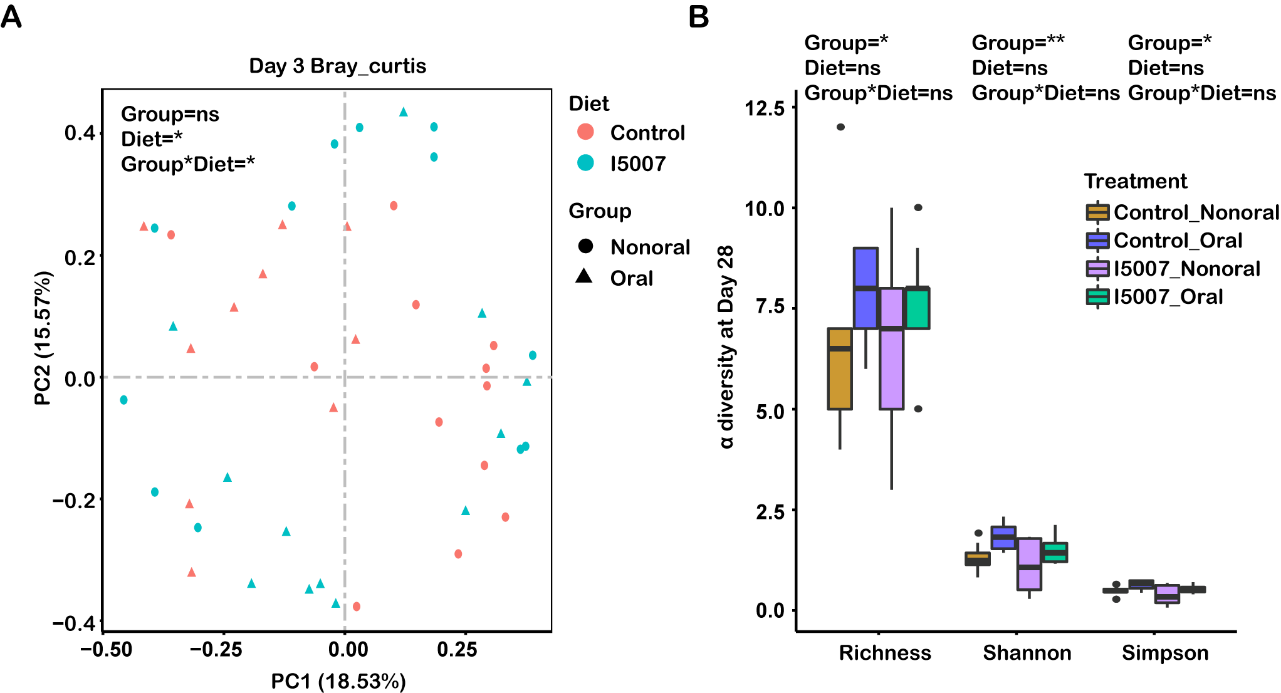

Supplement: Supplementary file 2 — Additional file 1: Figure S1. The effects of L. reuteri supplementation on the microbiota composition in the intestine of sows. (A) Dynamic changes in α-diversity (richness, Shannon and Simpson index) between two groups across different ages. The data are expressed as the mean ±SEM. Differences were analyzed by two-way ANOVA based on the Scheirer-Ray-Hare test. (B) PCoA plot based on the Bray–Curtis distance of microbiota composition. Significance was calculated using PERMANOVA. (C) Bray–Curtis dissimilarity between the L. reuteri I5007 and control groups across different ages. The median of the data is shown. Differences were analyzed by two-way ANOVA based on the Scheirer-Ray-Hare test. (D) Histogram showing the relative abundances of the five most abundant phyla in the intestine of sows over time. (E) Heatmap showing the genera that were significantly affected by supplementation with L. reuteri I5007 on a certain day. The data are expressed as the mean relative abundance (%) in each group. The Wilcoxon rank-sum test was used to analyze variation between two groups at the same time point. Diet indicates dietary supplementation with L. reuteri or not; group indicates the oral administration of L. reuteri or not. Figure S2. Alterations in the colostrum microbial composition. Histogram of the structural composition of the microbiota at the phylum level (top 6) (A) and the family level (top 10) (B) in the colostrum samples. Significance was measured using the Wilcoxon rank-sum test. The horizontal bars within the box represent the median. (C) The α-diversity of bacteria was measured using the richness, Shannon and Simpson indices. (D) PCoA plots of significantly different species based on Bray–Curtis distance. Figure S3. Significantly different species between the I5007 and control groups. Histograms of significantly altered species with the criteria LDA>2 and P<0.05 from metagenome data between the two groups. The color of the bar indicates enrichment of the species [file 40168_2022_1336_MOESM1_ESM.zip › Figure S14.docx]

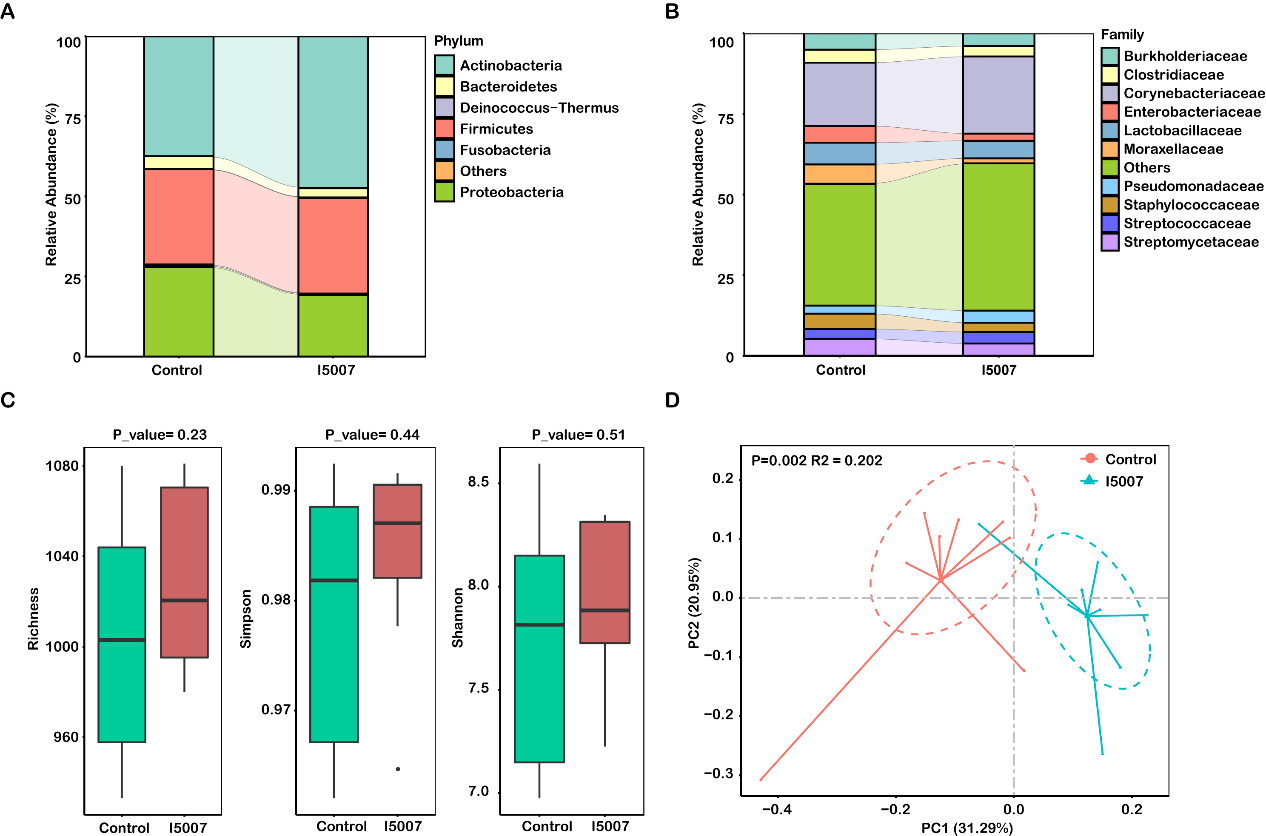

Supplement: Supplementary file 2 — Additional file 1: Figure S1. The effects of L. reuteri supplementation on the microbiota composition in the intestine of sows. (A) Dynamic changes in α-diversity (richness, Shannon and Simpson index) between two groups across different ages. The data are expressed as the mean ±SEM. Differences were analyzed by two-way ANOVA based on the Scheirer-Ray-Hare test. (B) PCoA plot based on the Bray–Curtis distance of microbiota composition. Significance was calculated using PERMANOVA. (C) Bray–Curtis dissimilarity between the L. reuteri I5007 and control groups across different ages. The median of the data is shown. Differences were analyzed by two-way ANOVA based on the Scheirer-Ray-Hare test. (D) Histogram showing the relative abundances of the five most abundant phyla in the intestine of sows over time. (E) Heatmap showing the genera that were significantly affected by supplementation with L. reuteri I5007 on a certain day. The data are expressed as the mean relative abundance (%) in each group. The Wilcoxon rank-sum test was used to analyze variation between two groups at the same time point. Diet indicates dietary supplementation with L. reuteri or not; group indicates the oral administration of L. reuteri or not. Figure S2. Alterations in the colostrum microbial composition. Histogram of the structural composition of the microbiota at the phylum level (top 6) (A) and the family level (top 10) (B) in the colostrum samples. Significance was measured using the Wilcoxon rank-sum test. The horizontal bars within the box represent the median. (C) The α-diversity of bacteria was measured using the richness, Shannon and Simpson indices. (D) PCoA plots of significantly different species based on Bray–Curtis distance. Figure S3. Significantly different species between the I5007 and control groups. Histograms of significantly altered species with the criteria LDA>2 and P<0.05 from metagenome data between the two groups. The color of the bar indicates enrichment of the species [file 40168_2022_1336_MOESM1_ESM.zip › Figure S2.docx]

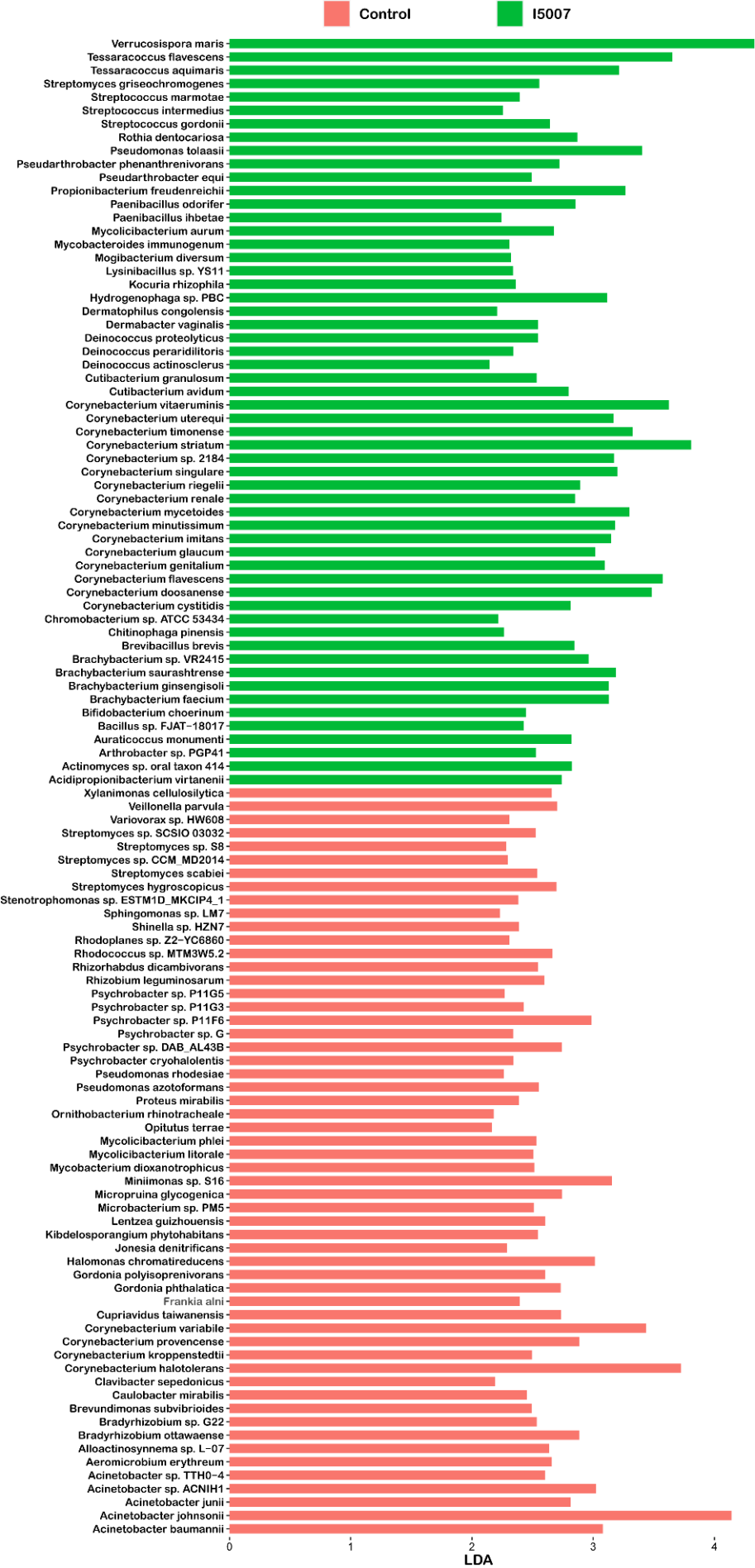

Supplement: Supplementary file 2 — Additional file 1: Figure S1. The effects of L. reuteri supplementation on the microbiota composition in the intestine of sows. (A) Dynamic changes in α-diversity (richness, Shannon and Simpson index) between two groups across different ages. The data are expressed as the mean ±SEM. Differences were analyzed by two-way ANOVA based on the Scheirer-Ray-Hare test. (B) PCoA plot based on the Bray–Curtis distance of microbiota composition. Significance was calculated using PERMANOVA. (C) Bray–Curtis dissimilarity between the L. reuteri I5007 and control groups across different ages. The median of the data is shown. Differences were analyzed by two-way ANOVA based on the Scheirer-Ray-Hare test. (D) Histogram showing the relative abundances of the five most abundant phyla in the intestine of sows over time. (E) Heatmap showing the genera that were significantly affected by supplementation with L. reuteri I5007 on a certain day. The data are expressed as the mean relative abundance (%) in each group. The Wilcoxon rank-sum test was used to analyze variation between two groups at the same time point. Diet indicates dietary supplementation with L. reuteri or not; group indicates the oral administration of L. reuteri or not. Figure S2. Alterations in the colostrum microbial composition. Histogram of the structural composition of the microbiota at the phylum level (top 6) (A) and the family level (top 10) (B) in the colostrum samples. Significance was measured using the Wilcoxon rank-sum test. The horizontal bars within the box represent the median. (C) The α-diversity of bacteria was measured using the richness, Shannon and Simpson indices. (D) PCoA plots of significantly different species based on Bray–Curtis distance. Figure S3. Significantly different species between the I5007 and control groups. Histograms of significantly altered species with the criteria LDA>2 and P<0.05 from metagenome data between the two groups. The color of the bar indicates enrichment of the species [file 40168_2022_1336_MOESM1_ESM.zip › Figure S3.docx]

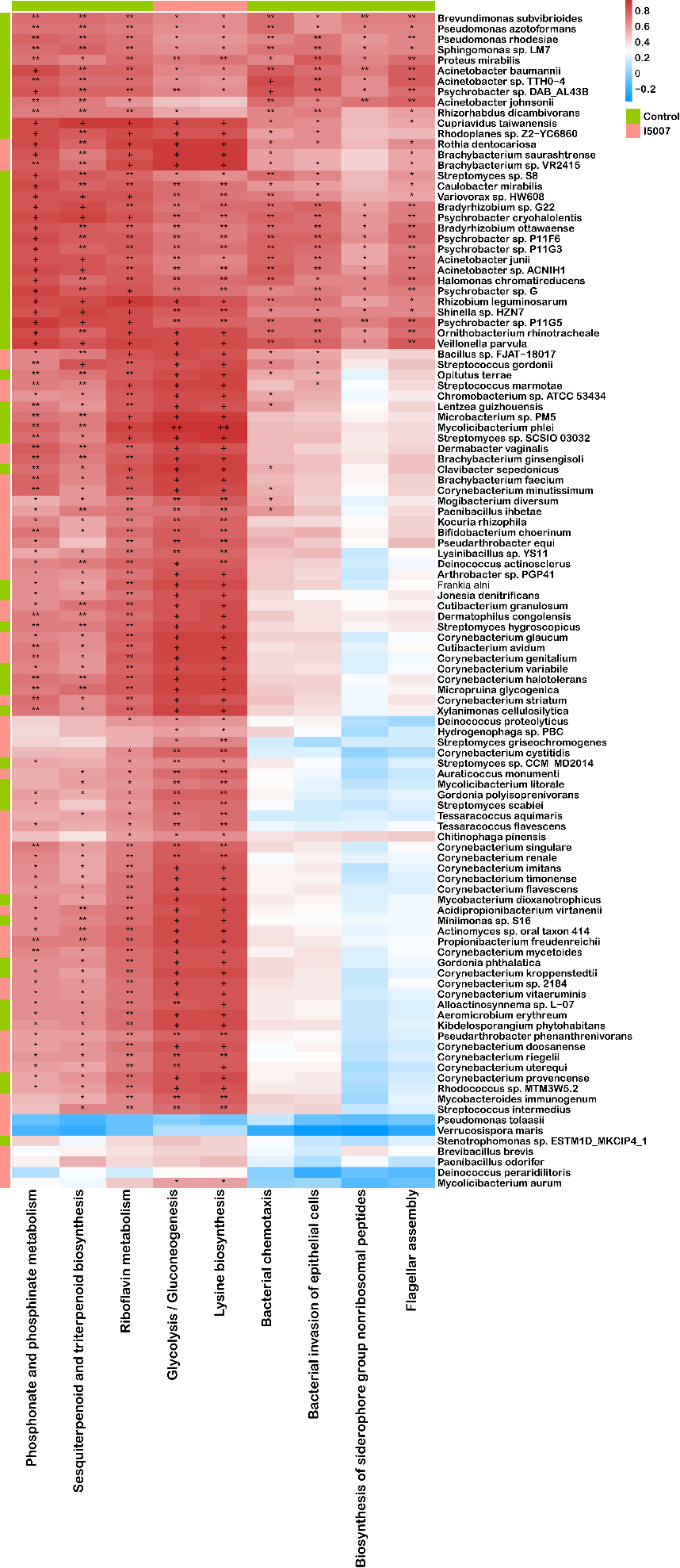

Supplement: Supplementary file 2 — Additional file 1: Figure S1. The effects of L. reuteri supplementation on the microbiota composition in the intestine of sows. (A) Dynamic changes in α-diversity (richness, Shannon and Simpson index) between two groups across different ages. The data are expressed as the mean ±SEM. Differences were analyzed by two-way ANOVA based on the Scheirer-Ray-Hare test. (B) PCoA plot based on the Bray–Curtis distance of microbiota composition. Significance was calculated using PERMANOVA. (C) Bray–Curtis dissimilarity between the L. reuteri I5007 and control groups across different ages. The median of the data is shown. Differences were analyzed by two-way ANOVA based on the Scheirer-Ray-Hare test. (D) Histogram showing the relative abundances of the five most abundant phyla in the intestine of sows over time. (E) Heatmap showing the genera that were significantly affected by supplementation with L. reuteri I5007 on a certain day. The data are expressed as the mean relative abundance (%) in each group. The Wilcoxon rank-sum test was used to analyze variation between two groups at the same time point. Diet indicates dietary supplementation with L. reuteri or not; group indicates the oral administration of L. reuteri or not. Figure S2. Alterations in the colostrum microbial composition. Histogram of the structural composition of the microbiota at the phylum level (top 6) (A) and the family level (top 10) (B) in the colostrum samples. Significance was measured using the Wilcoxon rank-sum test. The horizontal bars within the box represent the median. (C) The α-diversity of bacteria was measured using the richness, Shannon and Simpson indices. (D) PCoA plots of significantly different species based on Bray–Curtis distance. Figure S3. Significantly different species between the I5007 and control groups. Histograms of significantly altered species with the criteria LDA>2 and P<0.05 from metagenome data between the two groups. The color of the bar indicates enrichment of the species [file 40168_2022_1336_MOESM1_ESM.zip › Figure S4.docx]

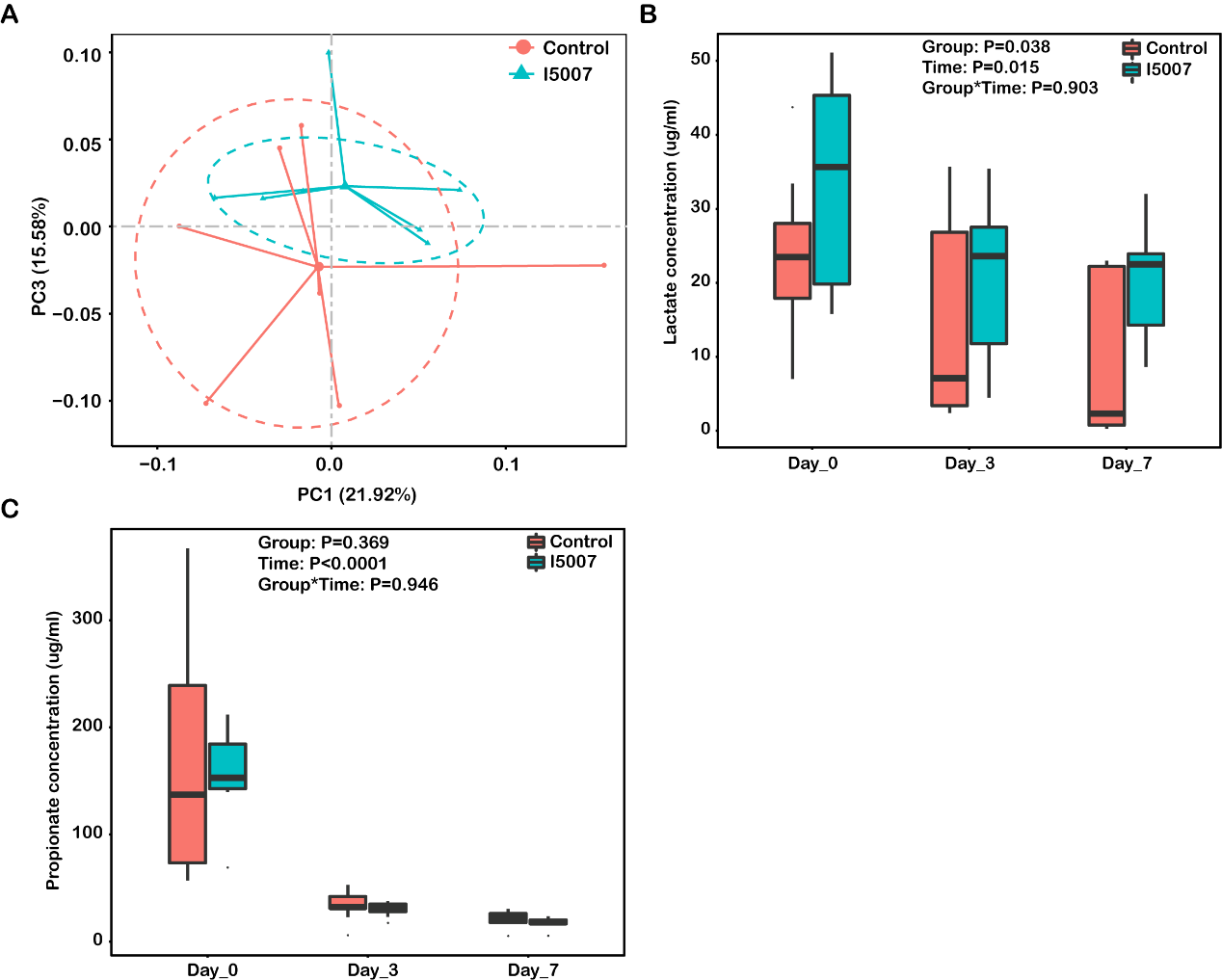

Supplement: Supplementary file 2 — Additional file 1: Figure S1. The effects of L. reuteri supplementation on the microbiota composition in the intestine of sows. (A) Dynamic changes in α-diversity (richness, Shannon and Simpson index) between two groups across different ages. The data are expressed as the mean ±SEM. Differences were analyzed by two-way ANOVA based on the Scheirer-Ray-Hare test. (B) PCoA plot based on the Bray–Curtis distance of microbiota composition. Significance was calculated using PERMANOVA. (C) Bray–Curtis dissimilarity between the L. reuteri I5007 and control groups across different ages. The median of the data is shown. Differences were analyzed by two-way ANOVA based on the Scheirer-Ray-Hare test. (D) Histogram showing the relative abundances of the five most abundant phyla in the intestine of sows over time. (E) Heatmap showing the genera that were significantly affected by supplementation with L. reuteri I5007 on a certain day. The data are expressed as the mean relative abundance (%) in each group. The Wilcoxon rank-sum test was used to analyze variation between two groups at the same time point. Diet indicates dietary supplementation with L. reuteri or not; group indicates the oral administration of L. reuteri or not. Figure S2. Alterations in the colostrum microbial composition. Histogram of the structural composition of the microbiota at the phylum level (top 6) (A) and the family level (top 10) (B) in the colostrum samples. Significance was measured using the Wilcoxon rank-sum test. The horizontal bars within the box represent the median. (C) The α-diversity of bacteria was measured using the richness, Shannon and Simpson indices. (D) PCoA plots of significantly different species based on Bray–Curtis distance. Figure S3. Significantly different species between the I5007 and control groups. Histograms of significantly altered species with the criteria LDA>2 and P<0.05 from metagenome data between the two groups. The color of the bar indicates enrichment of the species [file 40168_2022_1336_MOESM1_ESM.zip › Figure S5.docx]

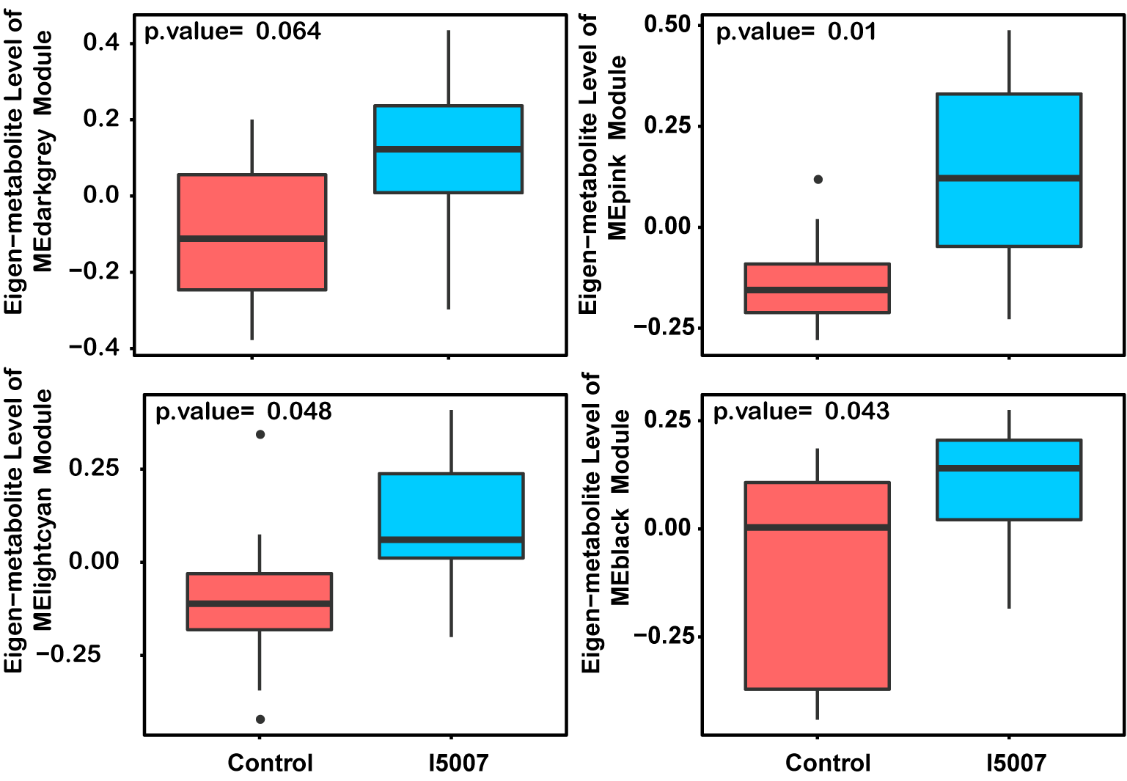

Supplement: Supplementary file 2 — Additional file 1: Figure S1. The effects of L. reuteri supplementation on the microbiota composition in the intestine of sows. (A) Dynamic changes in α-diversity (richness, Shannon and Simpson index) between two groups across different ages. The data are expressed as the mean ±SEM. Differences were analyzed by two-way ANOVA based on the Scheirer-Ray-Hare test. (B) PCoA plot based on the Bray–Curtis distance of microbiota composition. Significance was calculated using PERMANOVA. (C) Bray–Curtis dissimilarity between the L. reuteri I5007 and control groups across different ages. The median of the data is shown. Differences were analyzed by two-way ANOVA based on the Scheirer-Ray-Hare test. (D) Histogram showing the relative abundances of the five most abundant phyla in the intestine of sows over time. (E) Heatmap showing the genera that were significantly affected by supplementation with L. reuteri I5007 on a certain day. The data are expressed as the mean relative abundance (%) in each group. The Wilcoxon rank-sum test was used to analyze variation between two groups at the same time point. Diet indicates dietary supplementation with L. reuteri or not; group indicates the oral administration of L. reuteri or not. Figure S2. Alterations in the colostrum microbial composition. Histogram of the structural composition of the microbiota at the phylum level (top 6) (A) and the family level (top 10) (B) in the colostrum samples. Significance was measured using the Wilcoxon rank-sum test. The horizontal bars within the box represent the median. (C) The α-diversity of bacteria was measured using the richness, Shannon and Simpson indices. (D) PCoA plots of significantly different species based on Bray–Curtis distance. Figure S3. Significantly different species between the I5007 and control groups. Histograms of significantly altered species with the criteria LDA>2 and P<0.05 from metagenome data between the two groups. The color of the bar indicates enrichment of the species [file 40168_2022_1336_MOESM1_ESM.zip › Figure S6.docx]

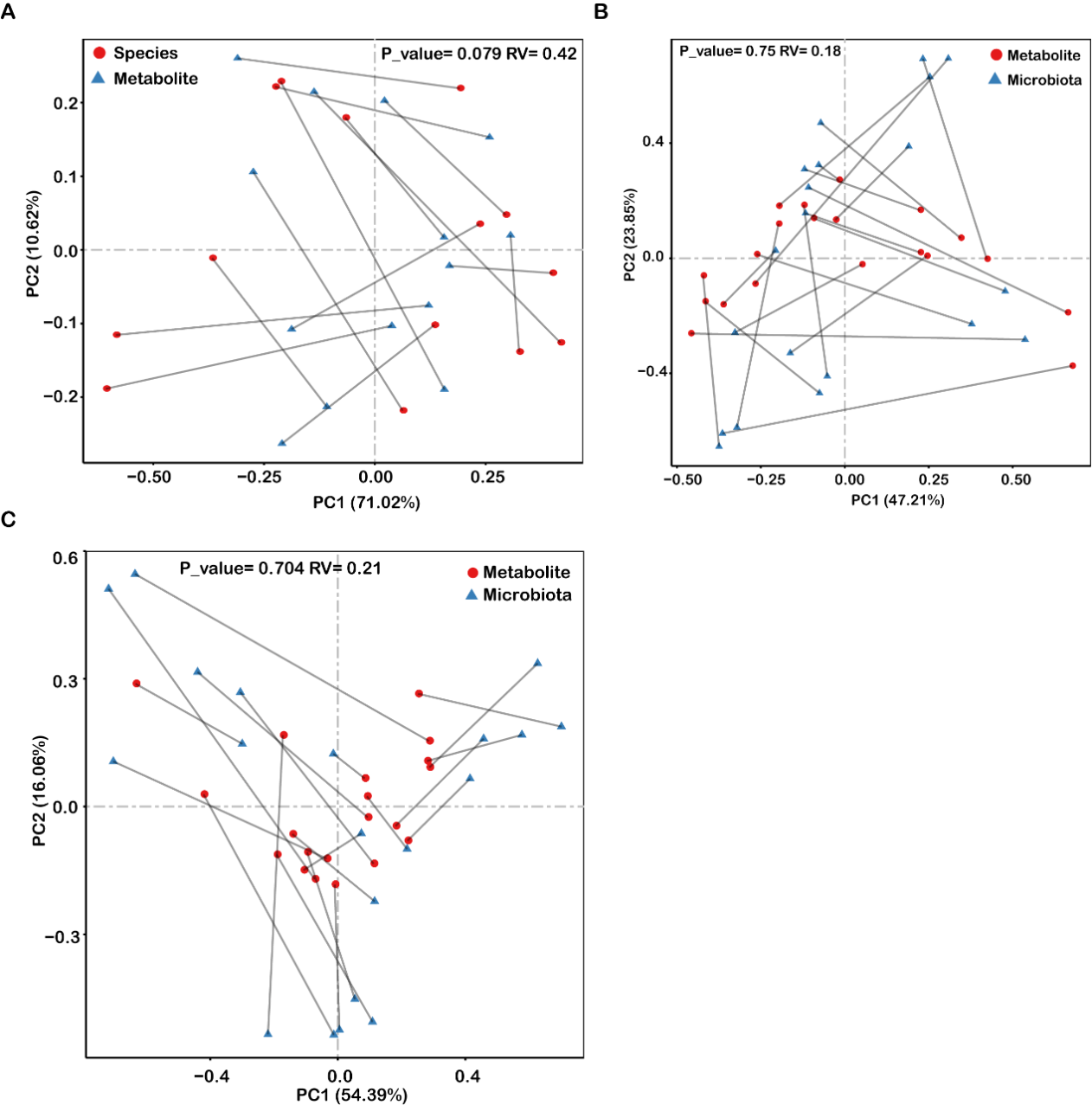

Supplement: Supplementary file 2 — Additional file 1: Figure S1. The effects of L. reuteri supplementation on the microbiota composition in the intestine of sows. (A) Dynamic changes in α-diversity (richness, Shannon and Simpson index) between two groups across different ages. The data are expressed as the mean ±SEM. Differences were analyzed by two-way ANOVA based on the Scheirer-Ray-Hare test. (B) PCoA plot based on the Bray–Curtis distance of microbiota composition. Significance was calculated using PERMANOVA. (C) Bray–Curtis dissimilarity between the L. reuteri I5007 and control groups across different ages. The median of the data is shown. Differences were analyzed by two-way ANOVA based on the Scheirer-Ray-Hare test. (D) Histogram showing the relative abundances of the five most abundant phyla in the intestine of sows over time. (E) Heatmap showing the genera that were significantly affected by supplementation with L. reuteri I5007 on a certain day. The data are expressed as the mean relative abundance (%) in each group. The Wilcoxon rank-sum test was used to analyze variation between two groups at the same time point. Diet indicates dietary supplementation with L. reuteri or not; group indicates the oral administration of L. reuteri or not. Figure S2. Alterations in the colostrum microbial composition. Histogram of the structural composition of the microbiota at the phylum level (top 6) (A) and the family level (top 10) (B) in the colostrum samples. Significance was measured using the Wilcoxon rank-sum test. The horizontal bars within the box represent the median. (C) The α-diversity of bacteria was measured using the richness, Shannon and Simpson indices. (D) PCoA plots of significantly different species based on Bray–Curtis distance. Figure S3. Significantly different species between the I5007 and control groups. Histograms of significantly altered species with the criteria LDA>2 and P<0.05 from metagenome data between the two groups. The color of the bar indicates enrichment of the species [file 40168_2022_1336_MOESM1_ESM.zip › Figure S7.docx]

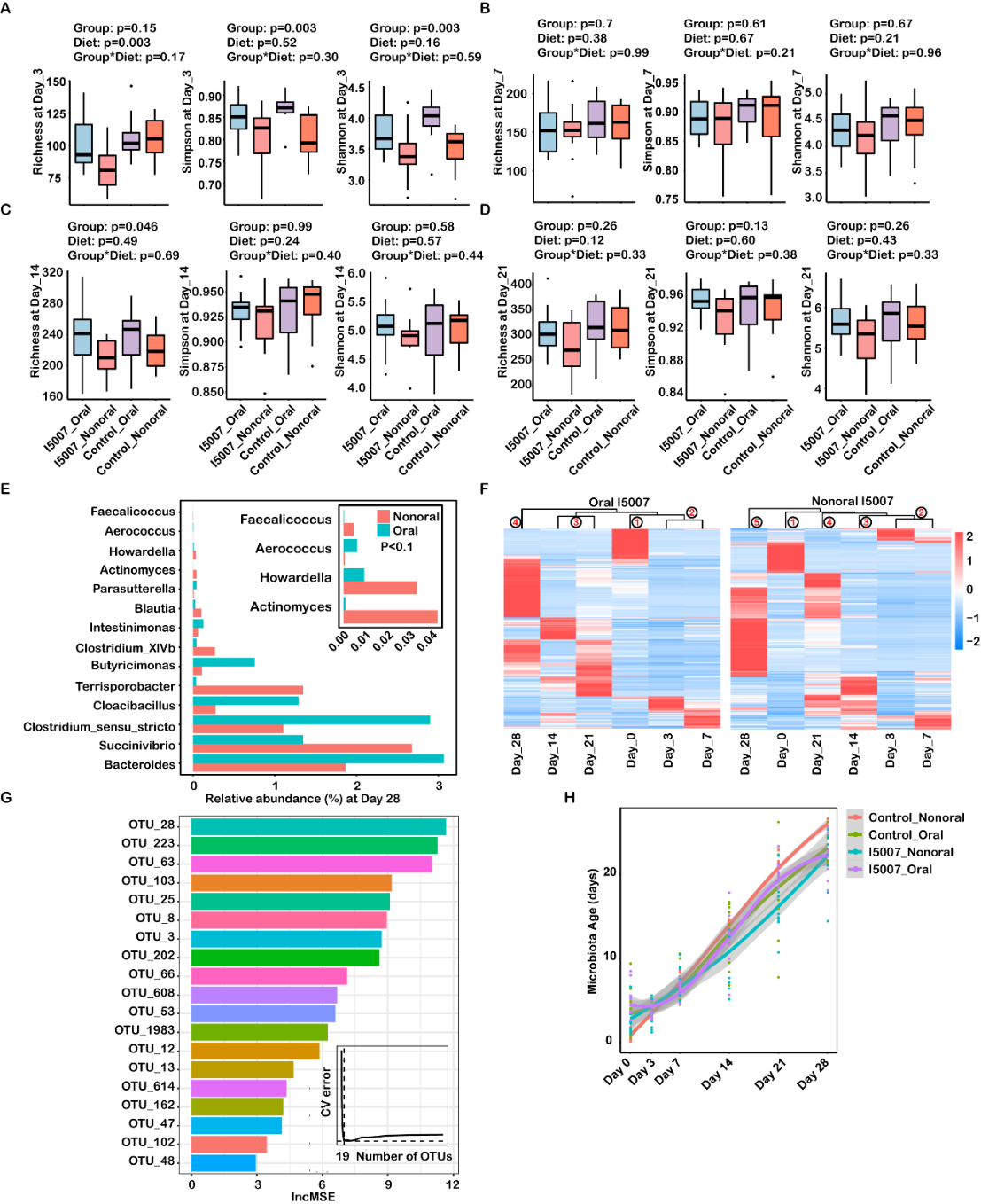

Supplement: Supplementary file 2 — Additional file 1: Figure S1. The effects of L. reuteri supplementation on the microbiota composition in the intestine of sows. (A) Dynamic changes in α-diversity (richness, Shannon and Simpson index) between two groups across different ages. The data are expressed as the mean ±SEM. Differences were analyzed by two-way ANOVA based on the Scheirer-Ray-Hare test. (B) PCoA plot based on the Bray–Curtis distance of microbiota composition. Significance was calculated using PERMANOVA. (C) Bray–Curtis dissimilarity between the L. reuteri I5007 and control groups across different ages. The median of the data is shown. Differences were analyzed by two-way ANOVA based on the Scheirer-Ray-Hare test. (D) Histogram showing the relative abundances of the five most abundant phyla in the intestine of sows over time. (E) Heatmap showing the genera that were significantly affected by supplementation with L. reuteri I5007 on a certain day. The data are expressed as the mean relative abundance (%) in each group. The Wilcoxon rank-sum test was used to analyze variation between two groups at the same time point. Diet indicates dietary supplementation with L. reuteri or not; group indicates the oral administration of L. reuteri or not. Figure S2. Alterations in the colostrum microbial composition. Histogram of the structural composition of the microbiota at the phylum level (top 6) (A) and the family level (top 10) (B) in the colostrum samples. Significance was measured using the Wilcoxon rank-sum test. The horizontal bars within the box represent the median. (C) The α-diversity of bacteria was measured using the richness, Shannon and Simpson indices. (D) PCoA plots of significantly different species based on Bray–Curtis distance. Figure S3. Significantly different species between the I5007 and control groups. Histograms of significantly altered species with the criteria LDA>2 and P<0.05 from metagenome data between the two groups. The color of the bar indicates enrichment of the species [file 40168_2022_1336_MOESM1_ESM.zip › Figure S8.docx]

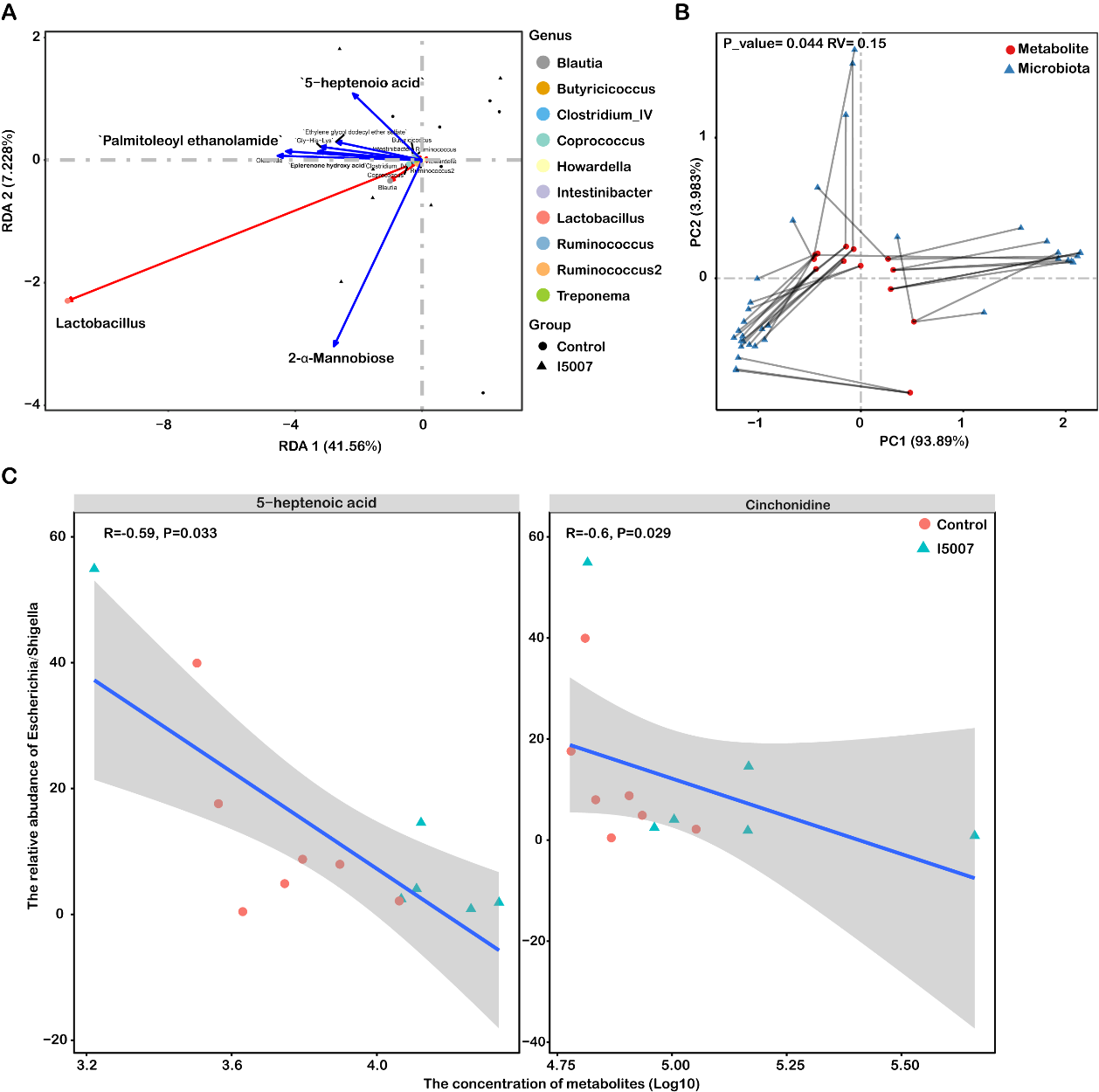

Supplement: Supplementary file 2 — Additional file 1: Figure S1. The effects of L. reuteri supplementation on the microbiota composition in the intestine of sows. (A) Dynamic changes in α-diversity (richness, Shannon and Simpson index) between two groups across different ages. The data are expressed as the mean ±SEM. Differences were analyzed by two-way ANOVA based on the Scheirer-Ray-Hare test. (B) PCoA plot based on the Bray–Curtis distance of microbiota composition. Significance was calculated using PERMANOVA. (C) Bray–Curtis dissimilarity between the L. reuteri I5007 and control groups across different ages. The median of the data is shown. Differences were analyzed by two-way ANOVA based on the Scheirer-Ray-Hare test. (D) Histogram showing the relative abundances of the five most abundant phyla in the intestine of sows over time. (E) Heatmap showing the genera that were significantly affected by supplementation with L. reuteri I5007 on a certain day. The data are expressed as the mean relative abundance (%) in each group. The Wilcoxon rank-sum test was used to analyze variation between two groups at the same time point. Diet indicates dietary supplementation with L. reuteri or not; group indicates the oral administration of L. reuteri or not. Figure S2. Alterations in the colostrum microbial composition. Histogram of the structural composition of the microbiota at the phylum level (top 6) (A) and the family level (top 10) (B) in the colostrum samples. Significance was measured using the Wilcoxon rank-sum test. The horizontal bars within the box represent the median. (C) The α-diversity of bacteria was measured using the richness, Shannon and Simpson indices. (D) PCoA plots of significantly different species based on Bray–Curtis distance. Figure S3. Significantly different species between the I5007 and control groups. Histograms of significantly altered species with the criteria LDA>2 and P<0.05 from metagenome data between the two groups. The color of the bar indicates enrichment of the species [file 40168_2022_1336_MOESM1_ESM.zip › Figure S9.docx]
